# Deciphering the safeguarding role of cysteine residues in p53 against H_2_O_2_-induced oxidation using high-resolution native mass spectrometry

**DOI:** 10.1038/s42004-024-01395-w

**Published:** 2025-01-15

**Authors:** Manuel David Peris-Díaz, Artur Krężel, Perdita Barran

**Affiliations:** 1 Michael Barber Centre for Collaborative Mass Spectrometry, Manchester Institute of Biotechnology, Manchester, UK; 2 Department of Chemical Biology, Faculty of Biotechnology, University of Wrocław, F. Joliot-Curie 14a, Wrocław, Poland

**Keywords:** Chemical biology, Chemical modification, Mass spectrometry, Proteomics

## Abstract

The transcription factor p53 is exquisitely sensitive and selective to a broad variety of cellular environments. Several studies have reported that oxidative stress weakens the p53-DNA binding affinity for certain promoters depending on the oxidation mechanism. Despite this body of work, the precise mechanisms by which the physiologically relevant DNA-p53 tetramer complex senses cellular stresses caused by H_2_O_2_ are still unknown. Here, we employed native mass spectrometry (MS) and ion mobility (IM)-MS coupled to chemical labelling and H_2_O_2_-induced oxidation to examine the mechanism of redox regulation of the p53-*p21* complex. Our approach has found that two reactive cysteines in p53 protect against H_2_O_2_-induced oxidation by forming reversible sulfenates.

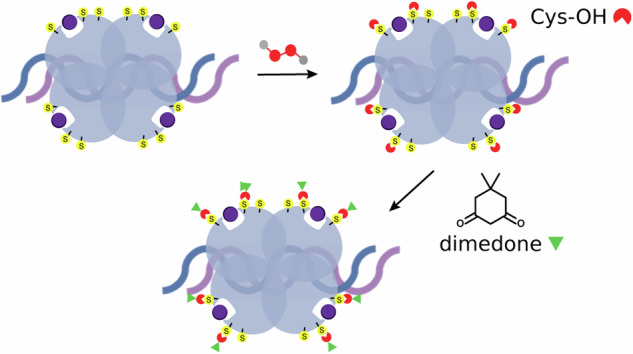

## Abstract (web summary)

Transcription factor p53 is sensitive and selective to a broad variety of cellular environments, with oxidative stress demonstrated to weaken p53-DNA binding for certain promoters, but the precise mechanisms by which the physiologically relevant DNA-p53 tetramer complex senses cellular stresses caused by H_2_O_2_ remain unknown. Here, native mass spectrometry and ion mobility–mass spectrometry are coupled with chemical labelling and H_2_O_2_-induced oxidation to probe the mechanism of redox regulation of the p53-*p21* complex.

## Introduction

The p53 protein is a transcription factor that regulates the expression of dozens of target genes related to cell cycle checkpoints, DNA repair, and apoptosis^1^. The human p53 protein contains 393 amino acids (aa) with four functional domains. The disordered N-terminal region (aa 1–42) is a transcriptional activation domain^2,3^. The C-terminal region contains an oligomerization domain (aa 323–356) and a regulatory domain (aa 360–393). In the central part of p53 is the sequence-specific DNA-binding domain (DBD, aa 91–289)^2,3^.

Biophysical studies have shown that the isolated p53-DBD can recapitulate properties of the full-length p53^4–6^. The DBD not only interacts with specific promoter response elements (RE), but also with many proteins and cofactors^7–10^. Approximately 50% of human tumours carry a mutation in *TP53*, of which 90% are missense mutations in the DBD-encoding sequence^11^. A consequence of these mutations is the impairment of the structure or the p53-DNA binding activity and/or specificity^4^. The function of p53 is strictly regulated in the cell by a variety of cellular and molecular mechanisms, such as post-translational modifications or long-range interactions between p53 domains^12–16^.

Hydrogen peroxide, the most important reactive oxygen species (ROS), has been shown to be capable of triggering both canonical DNA damage response (DDR) and oxidative signaling pathways, resulting in the transcriptional activation of p53 targets^17–22^. Early in vitro and in vivo studies have already shown that p53 binds to promoter sites in reducing conditions, but not in oxidizing conditions^23–25^. Uberti et al. concluded that, following H_2_O_2_ insult, oligodendroglia-like cells express the p21 protein in a p53-independent manner^20^. In H_2_O_2_-treated IMR-90 cells, Chen et al. found that p53 and p21 are induced by oxidative signaling rather than DDR pathways^26^. Barton group has reported that DNA-mediated charge transport, induced by ultraviolet radiation, promotes the oxidative dissociation of p53 from the DNA repair-associated *Gadd45* RE through an electron-based mechanism involving the oxidation of a cysteine residue (Cys277 or Cys275) and disulfide formation^22^. Similarly, Buzek et al. explored p53’s DNA binding in ultraviolet radiation-exposed WS-1 cells, showing its dissociation from the *Gadd45* sequence but remaining bound to the cell cycle-regulating *p21* RE. Site-directed mutagenesis experiments revealed that Cys277 was responsible for gene-selective transcription^18^.

Well established bottom-up mass spectrometry (MS)-based proteomics plays a major role deciphering protein composition, abundances, and post-translational modifications^27^. In the bottom-up MS approach, protein sequences are easily identified and quantified after enzymatic digestion into amenable smaller peptides, whose sequences are commonly determined by liquid-chromatography coupled to tandem mass spectrometry (LC-MS/MS)^28^. A body of work has shown that Cys oxidation in p53 itself may stabilize and activate p53 in vitro and in vivo. Using a bottom-up MS and a differential alkylation approach, it was discovered that Cys182 and Cys277 in the endogenous p53 of MCF7 and HCA2 cells were responsive to the thiol oxidant diamide^29^. Similarly, Barton and co-workers identified Cys277 and Cys141 as key residues  involved in the oxidative dissociation of the p53-DNA complex^22^, while Fersht and co-workers reported that Cys141 and Cys124 were the most reactive towards electrophiles^30^. Further studies have demonstrated that p53 can form intermolecular disulfides with 14-3-3θ and 53BP1 proteins involving Cys277^31^.

While bottom-up MS has been invaluable in identifying peptide-level modifications, it does not address proteins as intact entities. Proteins in biological systems exist as intact proteoforms, representing the diverse forms in which a protein product from a single gene can be found^32^. Today’s bottom-up MS technologies, which identify and quantify peptides derived from proteins rather than intact protein themselves, fail to provide protein-level information because the peptide-to-proteoform connectivity is lost^33^. In contrast to conventional MS-based proteomics, native MS intends to analyze intact proteins and their non-covalent complexes preserving the native-like state^34^. If combined with tandem mass spectrometry, this approach called native top-down is a powerful tool for higher-order structural characterization of protein complexes^35–39^. Upon such and many other successes, the past three decades have witnessed remarkable progress in native MS and established a new phase in structural biology^40–45^.

Consistently to previous reports, Langridge-Smith and co-workers using a top-down MS approach identified Cys182 and Cys277 as the most reactive Cys residues in DNA-free p53-DBD towards *N*-ethylmaleimide (NEM)^46^. Notwithstanding, the examination of the Cys reactivity against H_2_O_2_ in the DNA-p53 tetramer complex at the protein level remains unclear and is one of the aims of this study. For heterogeneous systems, measuring the masses of native complexes may not be sufficient to unambiguously identify structural information. In a seminal work by Clemmer and Jarrold, the capabilities of ion mobility-mass spectrometry (IM-MS) were demonstrated by revealing that a single charge state can present several conformations^47^. Since then, IM-MS has been employed to study protein systems and characterize their conformation and dynamics^48–52^. IM-MS is especially useful for analyzing heterogeneous samples and requires less sample preparation than other high-resolution techniques such as X-ray crystallography, NMR, and cryo-electron microscopy. Additionally, many systems that are not amenable to analysis using mentioned structural techniques can be interrogated by IM-MS. Thus, IM-MS has become an established method for investigating biomolecules^53–55^. IM-MS provides information about charge state distribution (CSD) and collision cross section (CCS). Proteins with larger CCS and/or extended conformations undergo a greater number of collisions with the buffer gas, leading to longer drift times or reduced mobility^56^. However, the resolution of the IM device may not be sufficient to distinguish closely related protein conformations. To aid in this purpose, gas-phase collisional activation (CA) can be used to probe subtle structural differences between similar conformations and study protein ion stability and dynamics^57^. For example, recording IM-MS experiments with increasing CA can be used to study unfolding mechanisms and estimate lab-frame unfolding energies^58–62^. These experiments, commonly referred to as collision-induced unfolding (CIU), have provided a practical approach with multiple applications such as uncovering ligand-based stabilization in TTR proteins^63^, characterization of biotherapeutic antibodies^64^ or metalloproteins^62,65^. A quantitative framework to determine activation energy of dissociation/unfolding has been proposed, providing a link between CIU and thermodynamic parameters^66^.

These conflicting results highlight the need for further experiments to clarify how the tumor transcription factor p53, in complex with DNA, responds to cellular stress induced by H_2_O_2_. To this end, we used hybrid MS approaches, including IM-MS, high-resolution native MS, bottom-up MS, and top-down MS. Following Cysteine alkylation, native MS was used to profile reactive Cys residues, and bottom-up/top-down MS identified them in both DNA-free and DNA-bound p53 states. We then monitored the H_2_O_2_ oxidation reaction on the DNA-p53 tetramer using native MS, and IM-MS was used to characterize the conformational space and monitor H_2_O_2_-induced structural changes. Dimedone, a probe that reacts with sulfenic acids, was then used to validate the H_2_O_2_ oxidation mechanism. Combining all this information, we showed how p53 can self-regulate and protect itself against H_2_O_2_ fluctuations using its Cys residues.

## Results

### Mapping the conformational landscape of p53 on DNA binding

**Fig. 1:**
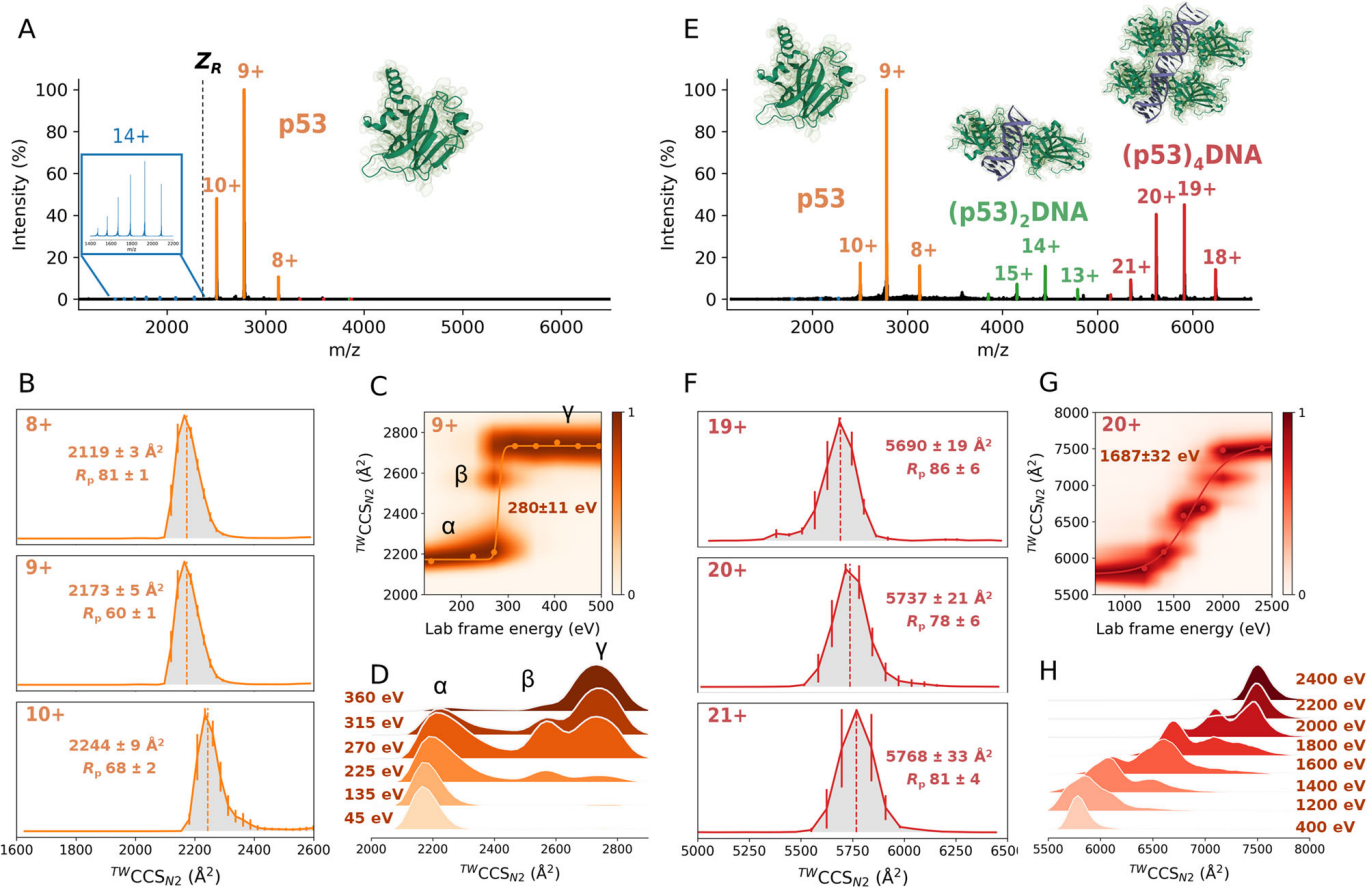
Ion mobility-mass spectrometry (IM-MS) experiments reveal a DNA-induced conformational change in p53, resulting in a more compact and less solvent-accessible conformation. Native mass spectra of WTp53 in the absence (**A**) and incubated with 0.25 equivalents of *p21* RE (**E**). Travelling wave (TW) ion mobility (IM)-derived collision cross sections (CCS) of quadrupole-selected charge states of WTp53 and (WTp53)_4_*p21* (**B**, **F**). The CCS values were calculated from three replicates, and the error bars plot along the CCS axis. Collision-induced unfolding (CIU) heat maps and CCS profiles for WTp53^9+^ (**C**, **D**) and (WTp53)_4_*p21*^20+^ (**G**, **H**). The PDBs used to illustrate monomer and tetramer complexes are 1OCJ and 3TS8, respectively.

The native mass spectrum of p53-DBD, referred to here as “p53” or “WTp53”, presents a bimodal CSD, suggesting the coexistence of at least two conformational families: one compact and globular, and the other more extended. In the absence of DNA, p53 is seen as a free monomer with one Zn(II) bound (Fig. 1A and Table S1). Previous studies have demonstrated that full-length p53 is capable of forming tetrameric complexes in the absence of DNA, as observed with an ultrastable quadruple mutant of p53 or full-length WT p53^67–69^. However, the inherent instability of full-length p53 makes it challenging to conduct experiments. Since isolated p53-DBD can recapitulate many physical properties of full-length p53, and most of the hot spot mutations are located in the DBD, it has become the preferred choice in structural studies^4–6^.

The charge state (*z*) region from 8 ≤ *z* ≤ 10 is assigned to a compact conformation that spans only three charge states, while the higher charge states (ranging 11 ≤ *z* ≤ 20), accounting for 2–5% of relative intensities, are assigned to forms presenting more disordered conformations. The Rayleigh limit (*Z*_*R*_) permits us to estimate the theoretical maximum number of positive charges that a globular protein can accommodate^70^, and it was found here to be 12. The Rayleigh limit also demarks the transition between compact and extended conformations (Fig. S1A).

To examine the conformers of p53 in more detail, we employed traveling wave ion mobility (TWIM)-MS. This allowed us to acquire arrival time distributions for each charge state, which were converted to ^TW^CCS_*N2*_ using a TWIMS calibration procedure. Extended conformations or ions with larger CCS undergo a greater number of collisions with the buffer gas and, as a result, have a longer drift time and reduced mobility than those that are more compact. The capability of IM-MS to separate conformational states has brought a powerful addition to the structural and dynamic interrogation of heterogeneous protein systems, which are not readily analyzed by other structural techniques^71–75^. TWIM-derived CCS distributions confirm that the four charge states with 8 ≤ *z* ≤ 11 populate a similar compact conformation, with ^*TW*^CCS_*N2*_ values spanning ~130 Å^2^ (Figs. 1B, S1 and Table S2). Comparing the experimental ^*TW*^CCS_*N2*_ values with the theoretical CCS (PDB: 1OCJ, 2792 Å^2^) calculated using the Projection Approximation using Rough Circular Shapes (PARCS) algorithm^76^ shows a slightly gas-phase collapse of the native-like p53 conformations, in agreement with previous data^68,77^. For ions with *z* > 11, multiple unfolded conformations are observed, and their size increases up to ~4000 Å^2^ for *z* = 18, which is consistent with previous measurements^9^.

Gas-phase activation of protein ions via CA can probe subtle structural differences between similar conformations, estimate ion stabilities and dynamics, and characterize unfolding mechanisms^78^. IM-MS under different CA conditions, recorded for mass-selected WTp53^9+^ ions, reveals a unfolding mechanism via a transition from the native compact α conformation with ^*TW*^CCS_*N2*_ ~ 2173 Å^2^ to an unfolded γ conformer with ^*TW*^CCS_*N2*_ ~ 2700 Å^2^, passing through a β intermediate conformation (Fig. 1C, D).

In the presence of DNA (*p21* RE), WTp53 forms a (Zn-p53)_4_DNA tetramer complex appearing at 40–50% relative intensity, most likely via (Zn-p53)_2_DNA dimer assembly, as this form is also observed (Fig. 1E). Monomeric disordered species are now present at levels lower than 1% of the relative intensity, suggesting that the remaining unbound p53 species are in a compact conformation. The conformational properties of the tetramer were examined by analyzing TWIM-derived CCS distributions of individual charge states, which showed a well-conserved single conformer with ^*TW*^CCS_*N2*_ ~ 5700 Å^2^ in all cases (Fig. 1F and Table S2). A close agreement was found between theoretical (PDB: 3TS8, 5644 Å^2^) and experimental CCS values for (Zn-p53)_4_DNA tetramer complex.

To compare the conformational diversity between monomeric and tetrameric species, we calculated the full width half maximum (∆^TW^CCS_N2_) (Table S2). The p53 monomer exhibits ∆^TW^CCS_N2_ ranging from 26 to 36 Å^2^, while the (p53)_4_DNA complex displays ∆^TW^CCS_N2_ values in the 67–74 Å^2^ range (Table S2). To understand the conformational changes of monomeric p53 upon DNA binding, we normalized the ∆^TW^CCS_N2_ to a single monomeric specie. When the ∆^TW^CCS_N2_ values of the (p53)_4_DNA complex are normalized to a single monomeric specie (∆^TW^CCS_N2_/n° subunits), they lead to ∆^TW^CCS_N2_ values of 16–19 Å^2^. As these values are much lower than the ∆^TW^CCS_N2_ values for p53 monomer (ranging from 26 to 36 Å^2^), we conclude that the monomer adopts a more compact conformation when bound to DNA. These results align with previous molecular dynamics simulations, which have reported a DNA-induced conformational change in p53, leading to a less solvent-accessible and compact conformation^10^. Specifically, these simulations identified that conformational changes in the L1 loop at the DNA-binding interface are coupled to shifts in S6-S7 loop. This regulatory mechanism of p53 upon DNA binding serves to protect p53 from interactions with partners that could potentially inhibit its transcription-dependent functions. Gas-phase CA of WT(p53)_4_DNA^20+^ ions reveals a complex multi-step unfolding mechanism (Fig. 1G, H), in contrast to the relatively simply unfolding process seen at the monomer level (Fig. 1C). We observe that the tetrameric DNA-p53 complex populate partially unfolded intermediates that are stable on the millisecond timescale. Atomic force microscopy studies have also revealed that binding to DNA alters the mechanical unfolding pathway, primarily due to the formation of hydrogen bonds with DNA, which leads to structural stabilization^79^.

To further support the idea that native MS and IM-MS can reproduce the structural features of p53, we performed identical experiments on one of the most common hot-spot mutations in p53, namely, R248Q^80^. The crystal structure of p53-DBD in complex with DNA reveals that R248 interacts with the minor groove of DNA, and therefore, R248Q is referred to as a DNA-contact mutant^3^. Our results show that R248Q exhibits a similar bimodal CSD in the native mass spectrum and a two-step mechanism with larger unfolding energies compared to the WT (Fig. S2). Furthermore, incubation of R248Qp53 with different stoichiometries of two RE, *p21* and *mdm2*, does not progress to the formation of the tetrameric DNA-p53 complex (Fig. S3). Altogether, this data, in agreement with previous results, demonstrates that the mutation disrupts p53-DNA binding properties and confirms that native MS can accurately reproduce not only the specific stoichiometry of the (Zn-p53)_4_DNA complex, but also retains the structural features observed *in solution*.

### Profiling reactive Cys residues in the (Zn-p53)_4_DNA complex

Cellular levels of p53 are tightly controlled by ubiquitination and proteasomal degradation^81^, and under cellular oxidative stress induced by ROS, p53 can be activated to regulate transcription of genes involved in cell cycle as *p21* or DNA repair as *Gadd45*, among others^17–22^. As cysteines are a preferred target for redox-linked regulatory modifications^82^, we profiled reactive Cys residues with native MS and chemical labelling with NEM. The p53 monomer contains ten Cys residues, from which three (Cys176, Cys238, and Cys242) together with His179 coordinate a structural Zn(II)^83^. Focusing on the isolated WTp53 first, two/three Cys react fast upon NEM incubation without alteration of the Zn(II) binding properties (Fig. S4A). As the reaction proceeds, up to five Cys residues are modified, forming Zn-p53(NEM)_5_ complex (Fig. S4B, C), suggesting that from the remaining five Cys residues, three coordinate Zn(II) and two are less accessible to the solvent. Then, we observed a cooperative modification mechanism whereby Zn(II) dissociates, and all of Cys residues were NEM-labelled leading to apo-p53(NEM)_10_ (Fig. S4D). In agreement with the structural role of Zn(II), the protein presents in higher charge states and more extended forms at this stage. Conflicting results have been published regarding the identification of reactive Cys in p53^21,22,29,46^. Fersht and co-workers performed Cys alkylation on p53-DBD and full-length p53 in the absence of DNA and determined by bottom-up MS that Cys141 and Cys124 were the most reactive^30^. Langridge-Smith and co-workers used NEM to label reactive Cys on DNA-free p53-DBD, and top-down MS identified Cys182 and Cys277 as NEM-labelled^46^. Barton and co-workers, using ultraviolet radiation, determined that Cys141 may be involved in the DNA-mediated oxidation DNA(*Gadd45*)-p53 complex^21^. In a later study, they reported that Cys277 is required for the oxidative dissociation of the p53-DNA complex and that mutation of Cys275 lowers p53’s affinity for DNA^22^. Taken together, these reports show how intricate is to attempt to unravel the mechanistic details of p53 oxidation at the molecular level.

**Fig. 2:**
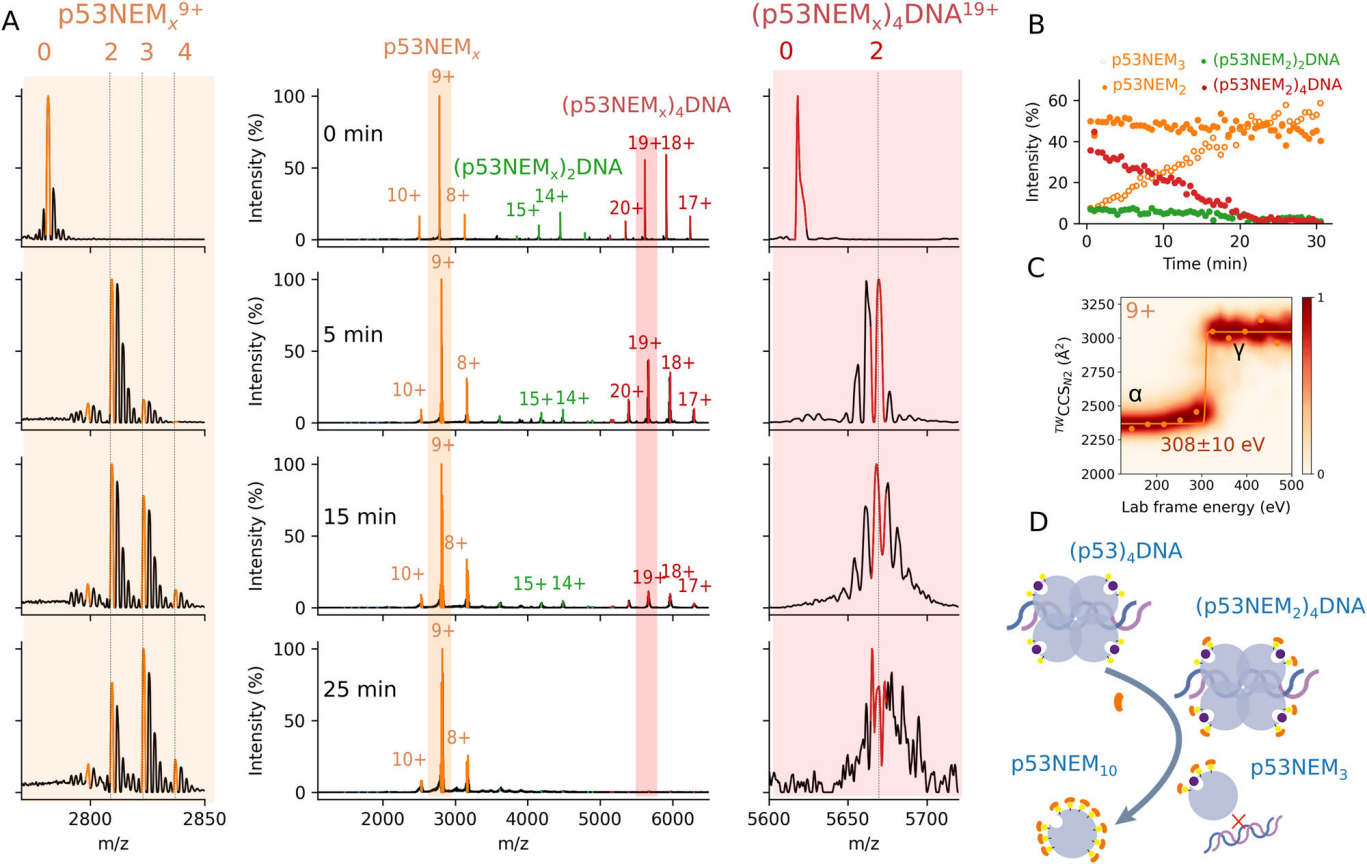
Native mass spectrometry identified two reactive Cys residues in the tetrameric p53-DNA complex. Native mass spectrum of WTp53 incubated with 0.25 equivalents of *p21* RE and 0.5 mM NEM recorded after 0, 5, 15, and 25 min of reaction (**A**). Plot of individual signal intensities as a function of the reaction time (**B**). Collision-induced unfolding (CIU) heat maps for quadrupole-selected p53NEM_2_^9+^ (**C**). Schematic representation of reaction mechanism (**D**). Purple and yellow spheres represent Zn(II) ions and S atoms from Cys residues, respectively. The orange moiety indicates a NEM modification.

Native mass spectrometry was used to examine the reactivity of the Cys residues in the (Zn-p53)_4_DNA complex formed upon DNA(*p21*) incubation (Fig. 2). As in the case of the free monomer, two Cys residues reacted fast upon NEM addition, and the p53NEM_2_ was able to form a DNA-p53 tetramer complex (Fig. 2A, B). CIU experiments for WTp53NEM_2_^9+^ revealed a CCS increase of ~150 Å^2^, a sharp unfolding transition with absence of β intermediate, and increased gas-phase stability (from 280 to 308 eV) than for WTp53^9+^ ions (Fig. 2C). Although these gas-phase stabilities are specific to the instrument and experimental conditions used^66^, it allowed us to estimate relative stabilities between ions of the same charge state.

Such a shift towards larger CCS values was also observed for the WT(p53NEM_2_)_4_DNA^19+/20+^ ions compared with WT(p53)_4_DNA^19+/20+^ (Fig. S5). This may suggest that NEM binds to surface-exposed Cys residues, resulting in an increased size and stability of the protein complex. Interestingly, binding the third NEM moiety to p53 almost completely disrupts p53-DNA binding, while Zn(II) remains bound to p53 (Fig. 2A). Further alkylation triggers Zn(II) dissociation and protein unfolding. From these results, we may conclude that two reactive Cys residues in p53 are partially involved in DNA binding, as evidenced by a 10–20% decrease in the relative intensity of the complex, and the third one completely abrogates p53-DNA complex formation. (Fig. 2D).

**Fig. 3:**
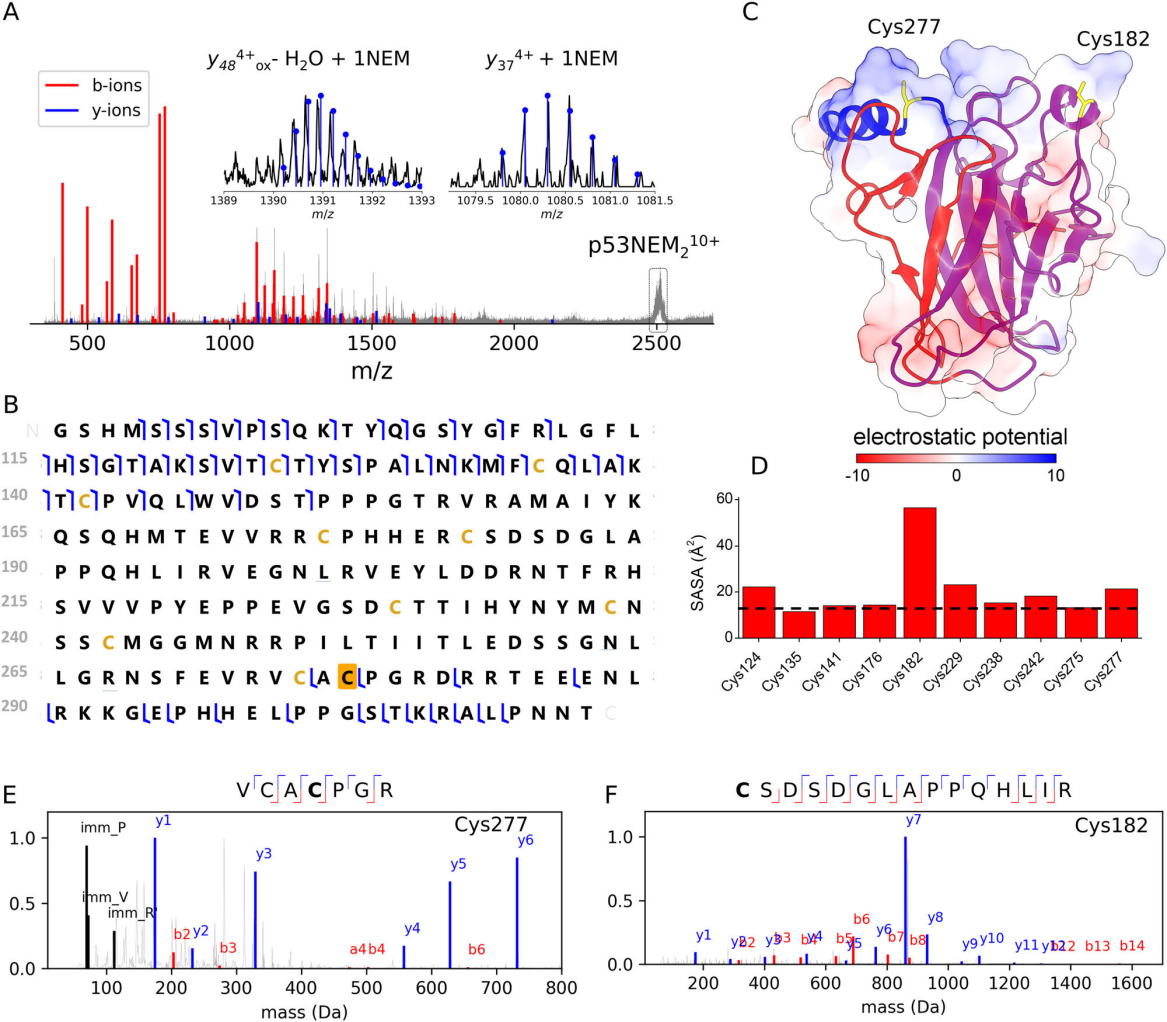
Top-down MS and bottom-up MS approaches identified Cys182 and Cys277 as the reactive Cys residues in p53. Native top-down mass spectrum of quadrupole-selected p53NEM_2_^10+^ ions obtained after incubation of the WTp53 with 0.25 equivalents of *p21* RE and 0.5 mM NEM for 5 min (**A**). Fragment location map from native top-down MS where the Cys277 modified by NEM is indicated by yellow (**B**). Electrostatic potential calculated for PDB:1OCJ (**C**). Solvent-accessible surface area (SASA) values for the Cys residues in p53 (**D**). The dashed line indicates the median SASA value. The MS/MS spectra supporting the annotation of NEM modification on Cys277 (**E**) and Cys182 (**F**). The Cys residues in p53 are coloured in yellow, and Cys NEM-labeled residues identified are marked in yellow in the peptide.

In order to localize the Cys residues harboring NEM, we performed native top-down MS experiments in which the intact protein ions were quadrupole-selected and subjected to collision-induced dissociation (CID) (Fig. 3A). We observe a CID spectrum mostly dominated by N-terminal b-fragment ions, where Cys124, Cys135, Cys141 residues were NEM-free (Fig. 3B). We found two characteristic y-fragment ions that localize Cys277-NEM labeled residue (inset Fig. 3A). Calculating the electrostatic potential shows that identified Cys277-NEM labeled lies in the positive electrostatic potential DNA-binding interface region (Fig. 3C). While electrostatic potential values provide valuable insights, other factors such as steric hindrance (bulkiness around the nucleophilic atom), solvent, charge and protein stability effects also influence nucleophilicity^84^. The Cys277 residue is located in the loop S10-H2 and shows higher solvent-accessible surface area (SASA) than median (Fig. 3D).

Considering previous data^18,22,29–31,85^, we can conclude that Cys277, a residue in close contact with DNA, is a reactive residue in DNA-bound p53 states although not critical for p53 function per se. Indeed, the results show that Cys277 alkylation did not lead to a complete abrogation of the tetrameric p53-DNA complex. Our data agree with in vitro cell studies that observed that the p53 C277S mutant was still transcriptionally activated^18^.

Using top-down MS, we did not localize the second NEM modification. However, considering that Cys124, Cys135, Cys141 residues were NEM-free and Cys176, Cys238, and Cys242 binds Zn(II), either Cys182, Cys229 or Cys275 was labeled by NEM (Fig. 3B). By employing a bottom-up MS approach, in which the protein is digested into peptides, and their masses are then measured, we found that Cys182 and Cys277 were labelled by NEM (Fig. 3E, F). The reactivity of Cys182 towards NEM can be rationalize by its high SASA values, allowing it to easily react with NEM (Fig. 3D). Our results are in agreement with Langridge-Smith and co-workers, that also identified Cys182 and Cys277 as the most reactive Cys residues in DNA-free p53-DBD^46^.

In an effort to reactivate p53 mutants, Cys alkylation has gained attention, and several small electrophiles have been developed^30,85^. Therefore, we tested the capabilities of NEM to reactivate R248Qp53, a mutant with impaired DNA binding properties. As in the case of WT-p53, two Cys were alkylated by NEM, and the fragments obtained by native top-down localize NEM in Cys277. Upon Cys residue alkylation, the protein did not form a complex with DNA, leading us to conclude that alkylation of the Cys277 residue impair p53-DNA binding properties rather than reactivate them (Fig. S6).

### Discerning the protective role of reactive Cys against oxidation

**Fig. 4:**
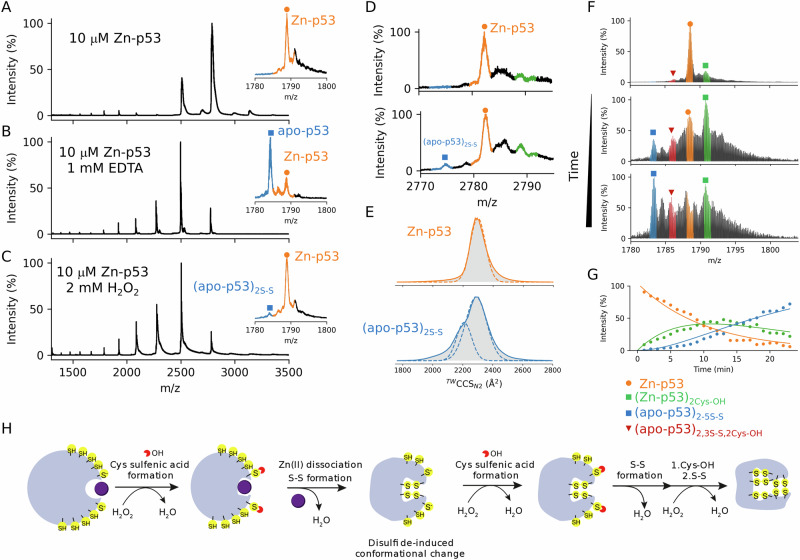
Native mass spectrometry characterized the H_2_O_2_ oxidation mechanism in p53. Native mass spectrum of WTp53 (**A**) incubated with 1 mM EDTA (**B**) or 2 mM H_2_O_2_ (**C**). IM-MS monitored the H_2_O_2_ oxidation of WTp53 over time (**D**). Travelling wave (TW) ion mobility (IM)-derived collision cross sections (CCS) of quadrupole-selected 9^+^ ions of Zn-p53 and (apo-p53)_2S-S_ (**E**) Calculated relative abundances from the mass spectrometry data in (**F**) and globally fitted to a two consecutive reactions A → B → C (**G**). Individual steps in the H_2_O_2_ oxidation mechanism of WTp53 (**H**). Purple and yellow spheres represent Zn(II) ions and S atoms from Cys residues, respectively.

Once we characterized the Cys residues reactivity in the DNA-p53 complex, we aimed to answer the following question: how does (Zn-p53)_4_DNA(*p21*) tetramer respond to an H_2_O_2_ insult? As abovementioned, several reports tried to address this issue, leading to ambiguous results mainly because it is not clear if the p21 protein is expressed in a p53-independent or dependent manner. Among ROS, H_2_O_2_ is an important intracellular messenger^86^. The moderate reactivity and high activation energies of the reaction with Cys residues results in a free H_2_O_2_ diffusion in the cell. As a consequence, it may reach long distances and target less surface-exposed proteins. In a first attempt, we incubated DNA-free p53 at increasing H_2_O_2_ concentrations and recorded a native MS spectrum after 5 min of reaction. Using a far beyond physiological H_2_O_2_/p53 molar ratios, H_2_O_2_ oxidized two thiolate anions to the sulfenate form (Cys-SO^−^, ∆m = 16 Da each one), which further reacted with excess H_2_O_2_ to yield two irreversible sulfinate species (Cys-SO_2_^−^, ∆m = 32 Da each one) (Fig. S7). Using an excess of ~200 molar equivalents of H_2_O_2_ to p53, which aligns with relative concentrations found in vivo^87^, sulfenic acid (Cys-SOH) formation was accompanied by the formation of disulfides, and any sulfinate species were detected (Fig. 4). Interestingly, the CSD shifted towards larger *z* and closely resembled that obtained for metal-free p53, albeit with Zn(II) remaining bound to p53 (Fig. 4A–C). This suggests a solution-phase conformational change altering SASA is induced by disulfide formation. Indeed, a second population with lower IM-derived CCS values was obtained for (apo-p53)_2S-S_^9+^ ions (where S-S stands for disulfide) than for Zn-p53, indicating that disulfide formation yields to more-compact and ordered conformations (Fig. 4D, E). As we were able to work under controlled oxidation conditions, we investigated the mechanism of oxidation by high-resolution native MS, which enabled real-time monitoring of the reaction (Fig. 4F, G). According to our results, the oxidation mechanism of Zn-p53 by H_2_O_2_ leads to the formation of bis-disulfides (apo-p53)_2S-S_ and proceeds by a stepwise mechanism (Figs. 4F and S8). Accurate mass measurements allowed us to conclude that the formation of bis-disulfides starts by the attack of two cysteine residues on two H_2_O_2_ molecules to yield two sulfenic acids ((Zn-p53)_2Cys-OH_), which reacts with Zn(II)-binding cysteine residues with concomitant Zn(II) dissociation and disulfide formation ((apo-p53)_2S-S_) (Fig. S8).

The formation of the disulfide product induces a conformational change, as evidenced by IM-MS. The observation of the (Zn-p53)_2Cys-OH_ intermediate is in good agreement with our NEM results that determined the presence of two reactive Cys residues in p53. The calculated relative abundances from the mass spectrometry data were globally fitted to two consecutive reactions A → B → C, where A is Zn-p53, B is (Zn-p53)_2Cys-OH_, and C is (apo-p53)_2S-S_ (Fig. 4). The observed first-order rate constants (*k*_obs_) were 0.09 s^−1^ and 0.08 s^−^^1^ at 523 K. Although qualitatively satisfactory, the data may not be directly compared with other experimental solvated data. We have based it on a single charge state, and we are then assuming this is equally representative of the protein in solution and that the ionization properties of the modified species are comparable during the calculation of relative abundances, and we ignore other factors (e.g., overlapping of isotopic distributions or estimation of S/N ratio), all of which are a potential source of errors that can alter the *k*_obs_ estimation. Nevertheless, it can be clearly seen that the concentration of Zn-p53 decreases exponentially while (Zn-p53)_2Cys-OH_ increases reaching a maximum value, and the product (apo-p53)_2S-S_ increased continuously (Fig. 4). As the reaction proceed, (apo-p53)_2S-S_ cooperatively reacts with H_2_O_2_ to yield (apo-p53)_5S-S_ via a sulfenic acid intermediate (apo-p53)_2S-S,2Cys-OH_ (Figs. S8 and 4G). To further confirm the formation of disulfides, aliquots of the reaction mixture were taken at various time points, and free Cys labelled with NEM (Fig. S9A–C). In the absence of H_2_O_2_, ten Cys residues were modified by NEM, indicating that all the Cys residues were in their reduced form (Cys-SH) (Fig. S9D). Upon H_2_O_2_ reaction, the species with 4, 6, and 8 NEM appeared, indicating that 6, 4, and 2 Cys residues were oxidized (Fig. S9E). Increasing H_2_O_2_ concentration resulted in the increase of apo-p53(NEM)_6_, suggesting a preference formation of a bis-disulfide product, as hypothesized in a previous report^88^.

**Fig. 5:**
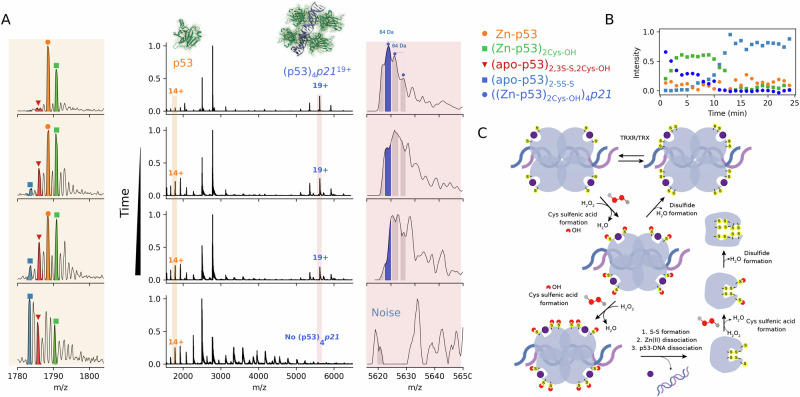
Time-resolved high-resolution native MS elucidated the H_2_O_2_ oxidation mechanism in the tetrameric DNA-p53 complex. Native mass spectrum of WTp53 pre-incubated with 0.25 equivalents of *p21* RE incubated with 2 mM H_2_O_2_ was monitored over time (**A**). Peaks were assigned for monomeric p53^14+^ species and for (p53)_4_*p21*^19+^. Calculated relative abundances from the time-resolved mass spectrometry data (**B**). Individual steps in the H_2_O_2_ oxidation mechanism of DNA-p53 tetramer complex (**C**). Initially, H_2_O_2_ oxidizes the ((Zn-p53))_4_DNA(*p21*) complex, forming Cys sulfenic acids (Cys-OH). Under physiological conditions, sulfenic acids in p53 react with non Zn(II)-binding cysteine residues, forming reversible disulfides that can be reduced by the buffer redox agents. Under oxidative conditions, when PRDX/TRX becomes oxidized, the complex undergoes oxidative dissociation. Here, sulfenic acids in p53 react with Zn(II)-binding cysteine residues, resulting in Zn(II) dissociation and disulfide formation, ultimately leading to dissociation from the promotor sequence. Zn(II), S atoms from Cys residues, and O atoms from H_2_O_2_ are represented by purple, yellow and red spheres, respectively.

Finally, we examine the H_2_O_2_ oxidation of the (Zn-p53)_4_DNA(*p21*) tetramer. Time-resolved measurements were used to monitor the relative intensities of the species involved in the reaction (Fig. 5). As in the prior experiment, two sulfenic acids are rapidly formed in both the DNA-free ((Zn-p53)_2Cys-OH_) and DNA-bound ((Zn-p53)_2Cys-OH_)_4_DNA(*p21*) p53 states (Fig. 5A–C). Moreover, the detection of signals with ∆m = 64 Da only in the DNA-bound complexes suggests either Cys oxidation in p53 or oxidative DNA damage, likely producing oxidized guanines (Fig. 5A). Guanine has the lowest oxidation potential compared to the other DNA bases and is therefore, the most readily oxidized base^89^. Indeed, biologically relevant 8-oxo guanine patterns have been mapped in human cells treated with physiological concentrations of H_2_O_2_, resembling two mutational signatures found in cancer^89^. Monitoring the mass distribution of the DNA-p53 complexes and monomeric p53 formation over time allowed us to calculate relative abundances and propose a plausible oxidation mechanism (Fig. 5C). The formation of ((Zn-p53)_2Cys-OH_)_4_DNA(*p21*) is followed by an oxidative dissociation, where the sulfenic acids in p53 react with Zn(II)-binding cysteine residues, resulting in concomitant Zn(II) dissociation and disulfide formation, which leads to dissociation from a promoter sequence for the *p21* gene (Fig. 5C). The (apo-p53)_2S-S_ species are eventually further oxidized by H_2_O_2_, yielding (apo-p53)_5S-S_. We next examined whether these mechanisms also occur in the presence of zinc metallothionein-2 (MT), a H_2_O_2_ scavenger^90^. The role of MT in redox processes is evidenced by the fact that its production increases in response to various oxidative stressors^91,92^. Additionally, cells that overexpress MT exhibit enhanced protection against oxidative stress^93–95^. Using 200:2:1 molar equivalents of H_2_O_2:_Zn_7_MT2:p53, we observed the rapid dissociation of six out of seven Zn(II) ions from MT2 and the formation of two sulfenic acids in Zn-p53 (Fig. S10A). Within 10 min of reaction with H_2_O_2_, all 20 cysteines of MT2 are oxidized, leading to the concomitant dissociation of all seven zinc ions (Fig. S10B). The sulfenic acids in p53 react as above with the formation of (apo-p53)_5S-S_. These results reflect the role of the two reactive Cys residues in protecting p53 against H_2_O_2_.

**Fig. 6:**
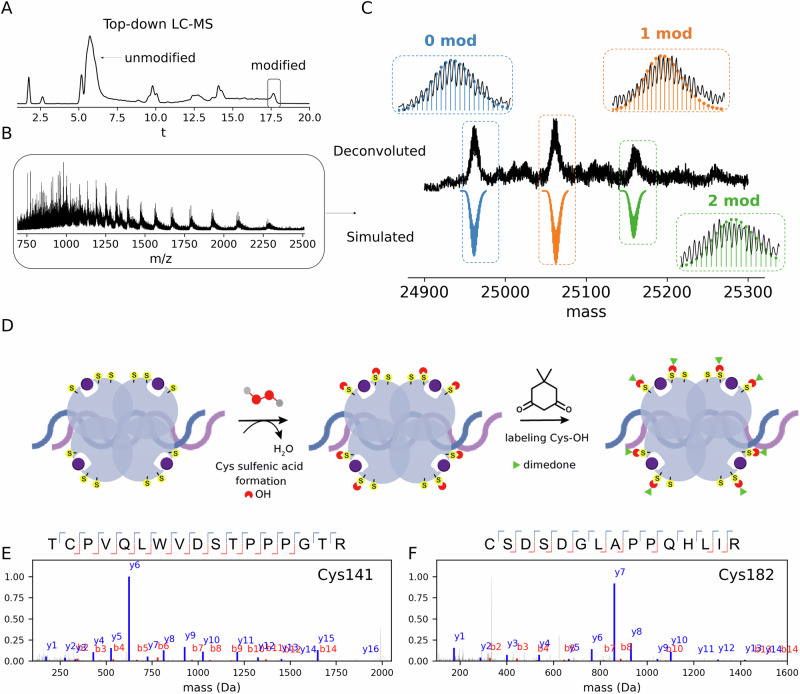
Sulfenic acid trapping with dimedone and top-down/bottom-up LC-MS experiments validated the reaction mechanism. Base peak chromatogram (BPC) from the intact LC-MS analysis of WTp53 reaction with H_2_O_2_ and incubated with dimedone (30 μM, 2 mM, and 5 mM concentrations, respectively) (**A**). Mass spectrum for the scans corresponding to the modified protein shown in the BPC (**B**). Deconvoluted spectrum and simulated isotopic distributions for the three species annotated (**C**). Schematic representation of the workflow used to trap sulfenic acids by dimedone (**D**). MS/MS spectra for the TCPVQLWVDSTPPPGTR^140–156^ peptide (**E**) and CSDSDGLAPPQHLIR^182–196^ peptide (**F**) that were identified with Cys141 and Cys182 dimedone-labeled, respectively.

To probe the existence of two sulfenic acids as intermediates in the H_2_O_2_ oxidation reaction and validate the reaction mechanism, we used dimedone, a compound that selectively traps sulfenic acids^90^. Direct injection of the reaction mixture resulted in unresolved charge state distributions, potentially leading to inaccurate mass assignments. For improved resolution and mass accuracy, the reaction mixture was separated and analyzed by a top-down liquid chromatography-mass spectrometry (LC-MS). The LC-MS chromatogram revealed the presence of two distinct populations: one composed of p53 molecules that did not react with H_2_O_2_, and the other comprising molecules that were oxidized and reacted with dimedone (Fig. 6A, B). Deconvolution of the mass spectrum and mass simulation revealed that two Cys residues reacted with dimedone, confirming that (Zn-p53)_2Cys-OH_ is a main intermediate in the H_2_O_2_ oxidation reaction (Fig. 6C, D). To localize these dimedone modifications, we employed a top-down and bottom-up MS approach. Intact MS/MS measurements did not lead to any informative fragment that can map the Cys modifications (Fig. S11). However, by using a bottom-up MS approach we were able to annotate peptides in which Cys141 and Cys182 were modified by dimedone (Fig. 6E, F). Peptides covering the Cys141 and Cys182 sequence were manually checked and showed nearly complete sequence coverage, validating the mapped modifications (Fig. 6E, F). In contrast to the nucleophilic addition reaction with NEM, Cys141, not Cys277, was the reactive residue towards H_2_O_2_. Redox-sensitive Cys residues are often deprotonated at physiological pH to undergo sulfenation^90^. In fact, the energy barrier for the oxidation of a thiol by H_2_O_2_ (~50 kcal∙mol^−^^1^) is two times higher than of a thiolate (~28 kcal∙mol^−1^)^91^. Calculations of the p*K*_a_ values for the Cys residues using PropKa analysis revealed that Cys182 (p*K*_a_ 7.55), but not Cys141 (p*K*_a_ 11), is an acidic cysteine residue. Considering the combined evidence from mass spectrometry and computational data, we can hypothesize that, owing its high SASA value (Fig. 3D) and acidic character, Cys182 in its thiolate form initiates the H_2_O_2_ oxidation reaction. The protein conformational changes induced by this reaction alter the protein microenvironment, lowering the Cys141 p*K*_a_ and triggering the oxidation cascade.

## Discussion

Different mechanisms are involved in the stabilization of p53 and the transcriptional activation of target genes responsible for DNA damage repair, cell cycle arrest, or apoptosis, by both redox signaling and DNA damage. Hydrogen peroxide, the most important ROS in redox signaling, has been shown capable of triggering both canonical DDR pathways and, through upstream ATM and stress-activated protein kinases JNK and p38MAPK pathways, leading to the phosphorylation and stabilization of p53^17^. A body of work has studied the effect of H_2_O_2_ on p53 showing that different mechanisms are involved in p53 stabilization and activation of target genes involved in DNA damage repair, cell cycle arrest, or apoptosis by redox signaling and DNA damage. For instance, early in vitro and in vivo reports have shown that p53 binds to promoter sites under reducing conditions but not under oxidizing conditions^23–25^. More recently, Barton and co-workers determined that p53 is likely irreversible oxidized when HeLa cells are incubated with millimolar H_2_O_2_ concentrations, although its transcriptional activity was not assessed^21^. In this sense, Uberti et al. showed that H_2_O_2_ induces a p53 nuclear translocation in oligodendroglia-like (OLN 93) cells and that they constitutively express p21 in a p53-independent manner^20^. However, it is not clear whether the observed activation of p53 by H_2_O_2_ is mediated through DDR, redox signaling, or both. Chen et al. addressed this question and found that H_2_O_2_-treated IMR-90 cells induced p53 and p21, while the levels of oxoG in DNA remained stable, indicating that DDR was not responsible for p53 and p21 activation^26^. Barton and co-workers, using electrophoretic mobility shift assays, determined that DNA-mediated charge transport (CT) can sequence-selectively promote the oxidative dissociation of p53 bound to the Gadd45 sequence, while p53 remains unaltered on the p21 promoter^21^. Using a bottom-up proteomics approach, they concluded that Cys141 forms a disulfide bond with an unlocalized Cys residue, promoting p53 dissociation from DNA. In a later work^22^, the authors employed a more sensitive mass spectrometry assay based on multiple reaction monitoring, which elucidated that Cys277 or Cys275, rather than Cys141, are involved in disulfide formation and p53 dissociation from DNA. Consistent with their findings, Buzek et al. using a bio-oligo pull-down DNA binding assay, investigated the DNA binding activity of p53 on ultraviolet-radiated WS-1 human fibroblast cells^18^. They found that p53 dissociates from the Gadd45 sequence but not from the p21 response element (RE), and that Cys277 is critical for the differential affinity of p53 to distinct REs.

Besides signaling pathways upstream of p53 or controlled by redox signaling, Cys oxidation by H_2_O_2_ in p53 itself has also been suggested to stabilize and activate p53. For instance, APR-249 (an alkylating reagent tested in a phase I/IIa clinical trial) reactivates mutant p53 by targeting Cys124 and Cys277^85^. Many other small molecules have been developed to reactivate mutant p53 proteins by covalently binding to cysteines, such as CP-31398, HO-3867,KSS-9, MIRA-1, PK11007, and STIMA-1. Several attempts have been made to identify the redox-active Cys residues in p53. Held et al. used a differential alkylation approach with the alkylating reagent NEM and found that Cys182 and Cys277 in endogenous p53 of MCF7 and HCA2 cells were sensitive to the thiol oxidant diamide^29^. However, the specific type of reversible cysteine oxidation remained to be determined.

Consistently, Scotcher et al. identified that Cys182 and Cys277 in monomeric p53 were reactive to the alkylating reagent NEM in vitro^46^. The metal binding status of p53 has also been linked to the sensitivity for Cys oxidation. Wu and Momand showed that at least one Cys residue per p53 molecule was highly sensitive to oxidation when MCF7 cells were treated with pyrrolidine dithiocarbamate (PDTC), a metal chelator^96^. PDTC treatment correlated with the oxidation state of Cys residues on p53 in vivo^97^. This body of work clearly indicates that Cys oxidation by H_2_O_2_ in p53 itself may play a role in p53 stabilization and activation in vivo. Despite being a subject of study for more than 25 years, yet it is not fully understood how Cys oxidation in p53 regulates p53 stability and function. This is partly due to the milieu of components involved, which hinders the isolation and study of Cys oxidation by H_2_O_2_ from DDR or redox signaling. This complexity is our rationale for conducting the study in vitro. When coupled with high-resolution native MS, we are able to resolve a chemical reaction that likely could not be done by other means due to the heterogeneity of the protein and reaction mixture.

A major concern and possible limitation of our conclusions may relate to the amount of H_2_O_2_ used in the experiments. Physiological H_2_O_2_ concentration is estimated to be buffered in the low nanomolar range, typically between 1 and 100 nM^98^. However, these estimations are based on quantifying extracellular H_2_O_2_, followed by calculating intracellular H_2_O_2_ concentration using a theoretical 100-fold extracellular-to-intracellular peroxide gradient^99^. Recent estimations, utilizing genetically encoded H_2_O_2_ probes like HyPer-3^99^, have reported even higher gradient concentrations, up to 650-fold^100^. However, it has been noted that there are intracellular spaces with even zero H_2_O_2_ concentration, which would make the gradient tend toward infinity^101^. Overall, rough estimations of the average basal H_2_O_2_ level and its fluctuations suggest different intracellular H_2_O_2_ concentrations, ranging from the nano to even picomolar range^102,103^. Under oxidative stress, however, H_2_O_2_ levels can increase up to 10,000 nM^98^. Our in vitro experiments used H_2_O_2_ concentrations much higher than physiological levels in absolute terms. Additionally, we employed a p53 concentration a million times higher than the reported physiological range (0.5–2 ng/ml)^104^. Considering that H_2_O_2_ is estimated to be between 10–100 nM and p53 between 0.005 and 0.04 nM, there is an excess of 250 to 20,000 molar equivalents of H_2_O_2_ to p53. In relative terms, we employed an excess of ~200 molar equivalents of H_2_O_2_ to p53, which aligns with relative concentrations found in vivo. Furthermore, our in vitro experiments appear to capture two different oxidation mechanisms. Using a far beyond physiological H_2_O_2_/p53 molar ratios, we observed that Cys oxidation rapidly forms two irreversible sulfinate species. However, using a seemingly reasonable H_2_O_2_/p53 molar ratios, the formed sulfenic acids do not react with an excess of H_2_O_2_ to form sulfinate species but instead react with free thiols to form disulfides—an oxidation product that can be reversed by the action of the redox buffers present in the cell, such as TRX or GRX.

There is a large body of work showing that oxidation of zinc finger transcription factors occurs both in vitro and in vivo. These include heat shock protein 33^105,106^, anti-sigma factor RsrA, protein kinase C and replication protein A^107^. In the above examples, H_2_O_2_ sensing occurs through the oxidation of the zinc-binding Cys residues. The zinc finger NEMO required for NF-kB activation is also target for H_2_O_2_, likely through Cys179 oxidation.^108^. H_2_O_2_ has also been demonstrated to oxidize cysteinyl thiols, inducing the formation of sulfenic acids and disulfide bonds in various protein targets such as arylamine N-acetyl transferase-1, indoleamine 2,3-dioxigenase, phospholipase A1, SUMO E1 UBA2 and PTP1B^109^ as well as the bacterial transcription factor OxyR^110^.

Another major question to consider is whether or not p53 can be oxidized in the nucleus in the presence of peroxidases, which contain catalytic Cys residues that react much faster than Cys residues in p53 with H_2_O_2_. Shi and Dansen excellently reviewed two PRDX models, the flood-gate model and the PRDX relay model^111^. Briefly, the PRDX model provides an explanation of how intrinsically unreactive protein thiols can be oxidized in response to low H_2_O_2_ concentration, as observed in proteomics studies. In this model, redox signaling starts at very low H_2_O_2_ concentration, oxidizing PRDX. Redox signaling gradually changes to damage signaling when PRDX becomes overoxidized and H_2_O_2_ is able to escape their scavenging function. Even though the intrinsic reactivity of the Cys residues in p53 is much lower compared to Cys residues in PRDX and TRX, as evidenced above, there is a large body of evidence that p53 itself is redox-sensitive in vivo.

The mechanism by which H_2_O_2_ regulates the stability and activity of the DNA-p53 complex through Cys residues in p53 itself remains unknown and was a motivation for our study. By applying ion mobility-mass spectrometry, we first characterized the gas-phase conformational landscape and structural properties of the p53 monomer, and DNA-p53 tetramer. Then, using NEM, we found that p53 contains two reactive Cys residues in both DNA-free and DNA-bound states. Using top-down and bottom-up MS approaches, we identified these residues as Cys277 and Cys182. Next, we monitored the H_2_O_2_ oxidation reaction on the DNA-p53 tetramer in real-time using native MS. Under physiologically relevant H_2_O_2_/p53 molar ratios, we observed that two cysteine residues attacked H_2_O_2_ molecules to yield two sulfenic acid intermediates per monomer. These intermediates later reacted with Zn(II)-binding cysteine residues, resulting in concomitant Zn(II) dissociation and disulfide formation, ultimately leading to the loss of transcriptional activity. To validate the proposed H_2_O_2_ oxidation mechanism, we used dimedone, a reagent that selectively reacts with sulfenic acids. We indeed observed the formation of two sulfenic acids, which were later localized to be formed at Cys141 and Cys182. Due to the high SASA value and acidic character of Cys182, we hypothesized that the mechanism starts with Cys182 attacking H_2_O_2_, accompanied by a conformational change that lowers the p*K*_a_ of Cys141 and triggers the oxidation cascade. Despite losing its transcriptional activity when oxidized, the apoprotein can be restored to its functional state under normal cellular conditions through the action of redox (including TRX, GRX) and metal buffers (such as metallothioneins, ZnT, and Zip zinc transporters). Initially, the disulfides can be reversed to thiols/thiolates by the action of TRX or GRX. Considering the cellular free Zn(II) concentration ranging from 10^−11^ to 10^−9^ M^112^, and the metal binding properties of p53 (*K*_d_ ~ 10^−^^15^ M)^113^, it is anticipated that reduced p53 will undergo remetallation. Our work evidences how p53 can self-regulate and defend itself against H_2_O_2_ fluctuations through its cysteine residues and reconciles the previously published data.

## Methods

### Expression and purification of WTp53, R248Qp53 and metallothionein

The expression vector pET15B encoding the human p53-DBD (residues 91-312) was a gift from Cheryl Arrowsmith (Addgene plasmid # 24866).^114^. Metallothionein-2 was overexpressed in *E. coli* cells (expression vector deposited in Addgene, plasmid ID 105693). The protein was overexpressed and purified in bacterial system as described in the *Supporting information*.

### Nanoelectrospray Ionization

All MS and IM-MS experiments were conducted using nanoelectrospray and samples prepared at 5–10 µM in 200 mM ammonium acetate (pH 6.8) and desalted using micro Bio-Spin 6 columns (Bio-Rad) prior to any experiment. The samples were then ionized from a borosilicate glass capillary (O.D. 1.2 mm, I.D. 0.9 mm, World Precision Instruments, Stevenage, UK) produced in-house using a Flaming/Brown P-1000 micropipette puller (Sutter Instrument Co., Novato, CA, USA). Ions were produced by applying a positive potential of 0.9–1.4 kV via a platinum wire (Goodfellow).

### High resolution native MS and top-down MS

MS experiments were performed on a Q-Exactive UHMR Orbitrap mass spectrometer (Thermo-Fisher Scientific, Bremen, Germany) equipped with nESI source. The spray voltage were in the 1–1.4 kV range, source temperature was set at 250 °C, and the S-lens RF was set at 100. No in-source trapping was applied. The ions were transported to the HCD cell with an injection energy of 10 eV where they were cool down by doubling the N_2_ gas pressure in the HCD cell (trapping gas pressure 2) compared to the standard value. Full MS scan data were acquired with the noise threshold parameter set to 3.64 at a set resolution of 12,000 and 200,000 at m/z 400. The native mass spectra was processed using Xcalibur 4.1 and in house Python 3.5 scripts.

Native top-down experiments were performed in a Synapt XS HDMS by quadrupole-selecting particular ions and increasing the trap collision energy. Data was analyzed using MassLynx v4.2 (Waters Corp., UK) and masses assigned using ProSight Lite^115^. Electrostatic surface was calculated by USCF Chimera^116^ using the PDB:1OCJ. Propka 2.0 was used to calculate p*K*_a_ values^117^.

### Bottom-up MS

To perform bottom-up MS experiments, N-ethylmaleimide- or dimedone-labelled p53 proteins were buffer exchanged to 100 mM ammonium bicarbonate. Trypsin (Sigma-Aldrich) was added at 1:1 (w/w), followed by the addition of 0.1% RapiGest SF (Waters Corp., UK) and overnight digestion at 37 °C. Samples were acidified to 0.5% formic acid (FA) to quench digestion, centrifuged, and desalted on Stage Tips eluting the peptides with 80:20 ACN:H_2_O 0.1% FA. After removing ACN by speed-vac, the peptides were resuspended in 0.1% FA. Samples were analyzed via LC-MS using a Waters Acquity UPLC mclass system coupled to a Synapt XS HDMS operated in a positive ion and resolution mode. Peptides were first trapped on a Waters Acquity BEH C18 1.7 µm VANGUARD column and then separated on a Waters Acquity UPLC BEH C18 1.7 µm, 1.0 · 100 mm. Mobile phase A consisted of 0.1% FA (v/v) in MilliQ water, while mobile phase B consisted of 0.1% FA (v/v) in ACN. The LC gradient was supplied at 40 µl·min-1 over 22 min gradient (5–35%B). Mass spectrometry analysis was performed using MSe data independent acquisition, which used a collision energy ramp from 20 to 45 V in a mass range of 50–200 m/z for the high energy scans. Raw LC-MSe data files were processed using ProteinLynx Global Server 3.0.3 (Waters Corp., UK) and searched against human p53 fasta protein sequence (UniProt P04637). The search was performed using 10 ppm as a precursor mass tolerance and a fragment mass tolerance of 5 ppm with three minimum fragment ion matches per peptide. Trypsin was set as the protease with a maximum of two missed cleavage allowed. Cysteine N-ethylmaleimide, cystine, and Cysteine dimedone were set as variable modifications. Leu-Enkephalin (556.27 m/z) was used as a lock mass. Peak lists were exported as text files and analyzed using custom scripts in Python 3.5.

### Ion Mobility-Mass Spectrometry

IM-MS experiments were carried out on a Synapt XS HDMS (Waters Corporation, Manchester, UK). The ion source was operated under gentle conditions to prevent ion activation (source temperature 30 °C, cone voltage 10 V, source offset 1). All of the experiments were carried out in sensitivity mode to maximize ion transmission at the expense of peak resolution. CIU experiments were performed by recording ion arrival time distributions under different trap collision energies in the 0–60 V range of a quadrupole-selected ions. Wave velocity and wave heights were set up at 300 ms^−1^ and 20 V, respectively. The helium cell and nitrogen traveling wave were operated at 200 and 75 ml min^−^^1^. The trap DC bias was set up at 35 V to minimize ion activation. The mass spectra were calibrated using 2 μg μl^−^^1^ NaI (1:1 water:isopropanol). Activation energies were reported as laboratory frame energy (*E*_lab_), accounting for the charge state of the mass-selected ion and are specific to Synapt XS HDMS and experimental conditions used^66^. Arrival time distributions were converted to ^TW^CCS_*N2*_ using a TWIMS calibration procedure^118^. Briefly, serum albumin (Bovine), concanavalin a (jack bean) and cytochrome C (equine heart) purchased from Sigma-Aldrich were dissolved in 200 AmAc and diluted to a 10 µM protein concentration, and the ATD recorded using identical parameters. Measurements were done in triplicate in different days to account for any source of variation, and data averaged. The literature CCS_N2_ values for the standards were obtained from A. P. France et al.^118^. CCS calibration was performed using IMSCal19 (Waters Corp., UK)^119^. Data were analyzed by means Masslynx v4.2 (Waters Corp., UK), ORIGAMI^120^, CIUSuite 2^57^, and custom Python 3.5 scripts.

### DNA oligonucleotides

All the oligonucleotides for DNA binding studies were synthesized by Sigma-Aldrich. The oligonucleotides encoding for the *p21* recognization element were 5′ F-GAACATGTCCCAACATGTTG-3′ and 5′ R-CAACATGTTGGGACATGTTC-3′. Olignucleotides encoding for the *mdm2* recgonition element were 5′F-GGGCTGGTCAAGTTGGGACACGTCCGGCGT3′ and 5′R-ACGCCGGACGTGTCCCAACTTGACCAGCCC-3′. Annealing was performed by heating the oligonucleotides at 95 °C for 10 min, and gradually cooling to 25 °C. The concentration of double stranded DNA used for the experiments was based on that all DNA was annealed, which to our purposes is accurate enough.

### Cysteine alkylation experiments

To profile reactive cysteine residues, 10 µM of either DNA-free (WTp53 and R248Qp53) or DNA-bound (WTp53) was incubated with 0.5–1 mM NEM at 25 °C. Aliquouts were withdrawn at 5, 15, 30, and 60 min and native mass spectrum or IM-MS was recorded.

### Oxidation experiments

Stock solutions of H_2_O_2_ were freshly prepared in miliQ H_2_O A set of reactions involving different experimental approaches were performed: (i) Isolated p53 monomer 10 μM (200 mM ammonium acetate) was incubated with 2 and 20 mM H_2_O_2_ for 5 min in ice. Samples were desalted using micro Bio-Spin 6 columns (Bio-Rad), loaded to in-house prepared needles and analyze using an Orbitrap UHMR mass spectrometer with a resolution set up at 200,000 @ *m/z* 400; (ii) Time course oxidation experiments of the isolated p53 monomer were performed by incubating 10 μM p53 (200 mM ammonium acetate) with 2 mM H_2_O_2_. The sample was loaded into the needle and the reaction was monitored using Orbitrap UHMR mass spectrometer operating at resolution 200,000 @ *m/z* 400, or Synapt XS HDMS.; (iii) A control experiment that shows the CSD shift was performed by incubating 10 μM p53 with 1 mM EDTA for 30 min at 25 °C. The sample was desalted using micro Bio-Spin 6 columns (Bio-Rad) and analyzed by native MS and IM-MS using Orbitrap UHMR mass spectrometer and Synapt XS HDMS.; (iv) 10 μM p53 was incubated with 0.25 molar equivalents of p21 for 10 min. Then, 2 mM H_2_O_2_ was added and the sample loaded in the needle. The reaction was monitored online by using an Orbitrap UHMR mass spectrometer, operating at resolution 12,000 @ *m/z* 400.; (v) 10 μM p53 was incubated with 0.25 molar equivalents of p21 for 10 min. Then, 20 μM of Zn_7_MT2 was added and incubated for 10 min. After this, 2 mM H_2_O_2_ was added and the sample loaded in the needle. The reaction was monitored online by using an Orbitrap UHMR mass spectrometer, operating at resolution 12,000 @ *m/z* 400.

### Alkylation and oxidation experiments

These experiments attempt to probe the formation of disulfides by labeling free Cys residues. In a first experiment, p53 monomer was incubated with 5 mM NEM (15 min, 25 °C, dark), the NEM excess removed by C18-Ziptip and analyzed under denaturing MS conditions using a Synapt XS HDMS. A second experiment p53 was first incubated with 2 or 5 mM H_2_O_2_ (5 min, 25 °C) and then 5 mM NEM was added and incubated for 15 min in 25 °C, dark. Sample later purified by C18-Ziptip and directly analyzed.

### Sulfenic acid trapping by top down LC-MS dimedone experiments

To probe the existence of sulfenic acids as intermediates during the oxidation reaction, 30 μM of p53 was incubated with 0.5–10 mM H_2_O_2_ in the presence of 5 mM dimedone (Sigma-Aldrich). Aliquots were taken after 15, 60, 120, and 300 min, and the reaction quenched by fivefold dilution and acidification (50:50 ACN:H_2_O 0.1% FA). Samples were analyzed via LC-MS using a Waters Acquity UPLC mclass system coupled to a Synapt XS HDMS operated in a positive ion and resolution mode. A 5 µl of sample was injected and proteins then separated on a Waters Acquity UPLC BEH C4 1.7 µm, 2.1 · 50 mm. Mobile phase A consisted of 0.1% FA (v/v) in MilliQ water, while mobile phase B consisted of 0.1% FA (v/v) in ACN. The LC gradient was supplied at 10 µl·min-1 over 20 min gradient (15–90%B). Full MS data was acquired in a range of 500–3000 m/z. Raw LC-MS data files were processed using Masslynx v4.2 (Waters Corp., UK) and Python 3.5 scripts.

### Kinetic analysis

The *m/z* spectrum was divided into two distinct populations: unfolded conformation ions (z > 11) and ions forming a native compact structure (z = 8–10). The choice of charge state 14 for the monomeric species was based on several considerations: first, the signals in the *m/z* region corresponding to 14+ were baseline resolved, allowing us to directly use the intensity for kinetic analysis. In contrast, the native charge states (8–10) exhibited an asymmetric baseline, and the peaks were not completely desolvated. Considering these factors, relying on their intensity could introduce errors. Additionally, ionization and transmission effects may vary to a further extent for different intermediates in native-like ions. To validate our approach, we performed similar kinetic analyzes with different ions from the unfolded population. We opted not to use deconvolution spectra, as they would incorporate the abovementioned source of errors. Regarding the ions corresponding to the p53-DNA complex, we chose the most intense ion 19+ since they exhibited similar transmission and desolvation effects. Thus, the intensities from the single charge state of the mass spectrum acquired in the experiment ii (Fig. 4F, G) were normalized to 0–1 on every scan and fitted with Eqs. (1–3), that correspond to two consecutive first-order reactions:

1 $$\left[{{\rm{A}}}\right]=[{{\rm{A}}}]_{0}\exp \left(-{k}_{1}t\right)$$

2 $$\left[{{\rm{B}}}\right]=\frac{[{{\rm{A}}}]_{0}{k}_{1}}{{k}_{2}-{k}_{1}}[\exp \left(-{k}_{1}t\right)-\exp \left(-{k}_{2}t\right)]$$

3 $$\left[{{\rm{C}}}\right]=[{{\rm{A}}}]_{0}-\left[{{\rm{A}}}\right]-[{{\rm{B}}}]$$

where [A]_0_ is the initial concentration of the reactant, *k*_1_ and *k*_2_ are the reaction constants and *t* is reaction time.

### Reporting summary

Further information on research design is available in the Nature Portfolio Reporting Summary linked to this article.

## Supplementary information

Transparent Peer Review file

Supplementary information

Reporting Summary

## Data Availability

The mass spectrometry data have been deposited to Figshare repository (10.6084/m9.figshare.25352884).
