# Supplementary material for: Deciphering the safeguarding role of cysteine residues in p53 against H2O2-induced oxidation using high-resolution native mass spectrometry
Source: Commun Chem. 2025 Jan 15;8:13. doi: 10.1038/s42004-024-01395-w (PMC11736120; doi:10.1038/s42004-024-01395-w)
Supplement: Supplementary file 2 — Supplementary information [file 42004_2024_1395_MOESM2_ESM.pdf]

Deciphering the safeguarding role of cysteine residues in p53 against H_2O_2-induced oxidation using high-resolution native mass spectrometry

Supporting Information

Manuel David Peris-Díaz [ab], Artur Krężel[b], Perdita Barran[a]

[a]*Michael Barber Centre for Collaborative Mass Spectrometry, Manchester Institute of Biotechnology, 131 Princess Street, Manchester, M1 7DN, United Kingdom*
[b]*Department of Chemical Biology, Faculty of Biotechnology, University of Wrocław, F. Joliot-Curie 14a, 50-383 Wrocław, Poland*

Experimental section

Expression and purification of WTp53 and R248Qp53. The expression vector pET15B encoding the human p53-DBD (residues 91-312) was a gift from Cheryl Arrowsmith (Addgene plasmid # 24866) [1]. The genetic construc encoded a fusion protein with N-terminal His_6-tag. Expression vectors were transformed into BL21(DE3)-RIL *E. coli* cells and cultured in LB media at 37°C until 0.8 OD_{600}. Cells were induced with 0.1 mM isopropyl-β-D-1-thiogalactopyranoside (IPTG) supplemented with 0.1 mM $ZnCl_2$ and incubated overnight at 16°C. After induction, the cells were harvested by centrifugation (4,000 × *g* for 20 min), the pellet resuspended in lysis buffer (20 mM Tris-HCl, pH 8.0, 500 mM NaCl, 100 µM $ZnCl_2$, 1 mM TCEP, 10 mM imidazol) and sonicated for 15 min with 30 s pulses. TCEP was used as is a weakly Zn(II) binding reducing agent [2]. This was followed by centrifugation (20,000 × *g* for 45 min). The supernatant was loaded into a HisTrap HP column (Sigma-Aldrich) using the FPLC pump from AKTA pure system with a 1 ml/min flow rate. The column was then washed with five column volumes with a binding buffer (20 mM Tris-HCl, pH 8.0, 500 mM NaCl, 1 mM TCEP, 10 mM imidazol) and the protein eluted with a 100 ml gradient from 10 to 500 mM imidazole by using a 3 ml/min flow rate. The fractions containing the fusion protein were assessed by SDS-PAGE and pooled. Imidazol was removed by purifying the protein in a PD-10 desalting columns

(GE Healthcare) equilibrated with purification buffer (20 mM Tris-HCl, pH 7.4, 150 mM NaCl, 1 mM TCEP). The His_6-tag was removed by overnight incubation with 10 units of thrombin at room temperature. The protein was concentrated using Amicon Ultra-4 Centrifugal Filter Units with a membrane cut-off of 10 kDa (Merck Millipore, USA) and subsequently purified on a size exclusion chromatography (Superdex 200 10/300 GL., GE Healthcare) equilibrated with purification buffer using an AKTA pure system. The protein concentration was determined from A_{280} using the extinction coefficient ε = 17130 M^{-1} cm^{-1} and the metal content determined by PAR assay [3-4]. The protein was oxidized by DTNB and the Zn(II) released measured by PAR (492 nm, 71 500 $M^{-1}cm^{-1}$). The native protein purified with ~ 0.96 Zn(II) equivalent. The mutation was prepared by site-directed mutagenesis (QuikChange II, Agilent) according to the manufacturer's instructions. The R248Qp53 mutant was expressed and purified as describe above.

Expression and purification of metallothionein. The expression vector (Addgene plasmid ID 105693) was transformed into BL21(DE3) *E. coli* cells and grown in a culture medium (1.1% tryptone, 2.2% yeast extract, 0.45% glycerol, 1.3% K_2HPO_4, 0.38% KH_2PO_4) at 37°C until an OD600 of ~0.5 was reached. Protein expression was induced by adding 0.1 mM IPTG to the cells, followed by overnight incubation at 20°C with vigorous shaking. All subsequent steps were performed at 4°C. The cells were centrifuged (4,000 × g for 10 min) and resuspended in 50 mL of cold buffer A (20 mM HEPES, pH 8.0, 500 mM NaCl, 1 mM EDTA, 1 mM TCEP). The cells were then sonicated for 30 minutes (1-minute cycles) and centrifuged (20,000 × g for 15 min). The expressed protein was purified using chitin resin. After centrifugation, the supernatant was incubated overnight with 20 mL of chitin resin in buffer A, then washed with 50 mL of buffer A and cleaved by adding 100 mM DTT. The resin was incubated for 48 hours at room temperature on a rocking platform. The eluted solution from the chitin column was acidified to pH ~2.5 with 7% HCl and concentrated using Amicon Ultra-4 Centrifugal Filter Units with a 3 kDa membrane cutoff (Merck Millipore, USA). The protein was then further purified using a size exclusion chromatography (SEC) SEC-70 gel filtration column (Bio-Rad) equilibrated with 10 mM HCl to obtain metal-free protein (apoMT2). The identity of the eluted protein from SEC was confirmed by ESI-MS using a Bruker Maxis Impact (Bruker Daltonik GmbH, Bremen, Germany) calibrated with a commercial ESI-TOF Tuning mix (Sigma-Aldrich). Thiol concentration was determined spectrophotometrically using a DTNB assay, and the Zn^{2+}

binding capacity was confirmed spectrophotometrically by Zn^{2+} and Cd^{2+} titrations [3-4]. To the collected fraction of purified thionein-2, a 10 molar excess of $ZnSO_4$ was added under a nitrogen blanket, and the pH was adjusted to 8.6 with 1 M Tris base. Samples were concentrated with Amicon Ultra-4 Centrifugal Filter Units with a 3 kDa membrane cutoff (Merck Millipore, USA) and subsequently purified using an SEC-70 gel filtration column (Bio-Rad) equilibrated with 20 mM Tris-HCl buffer at pH 8.6. Concentrations of thiols and Zn^{2+} were determined spectrophotometrically using DTNB and PAR assays, respectively [3-4].

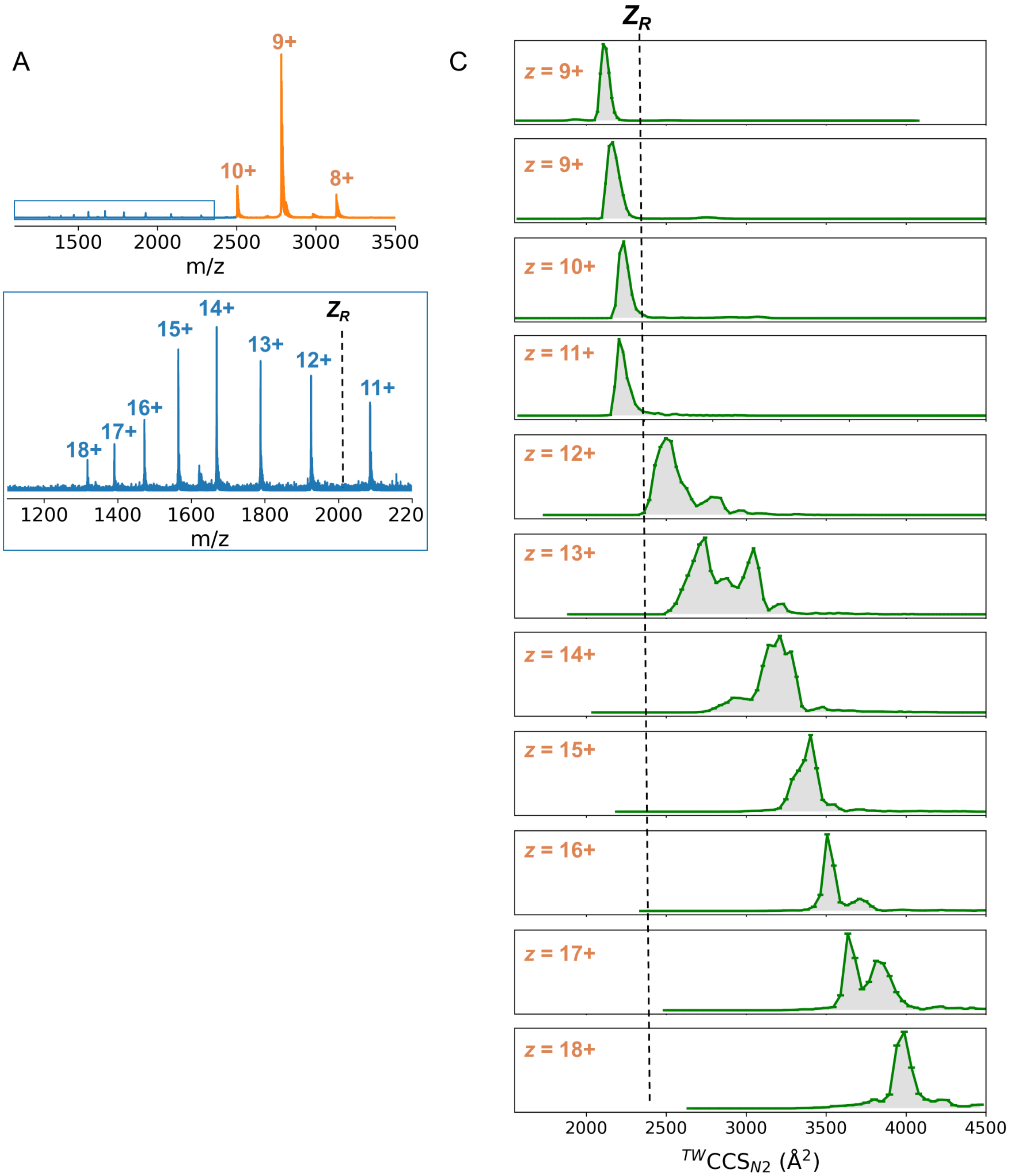

Figure S1. Native mass spectra of WTp53 (A) and IM-derived CCS distributions for different charge states. Z_R denotes the Rayleighn limit.

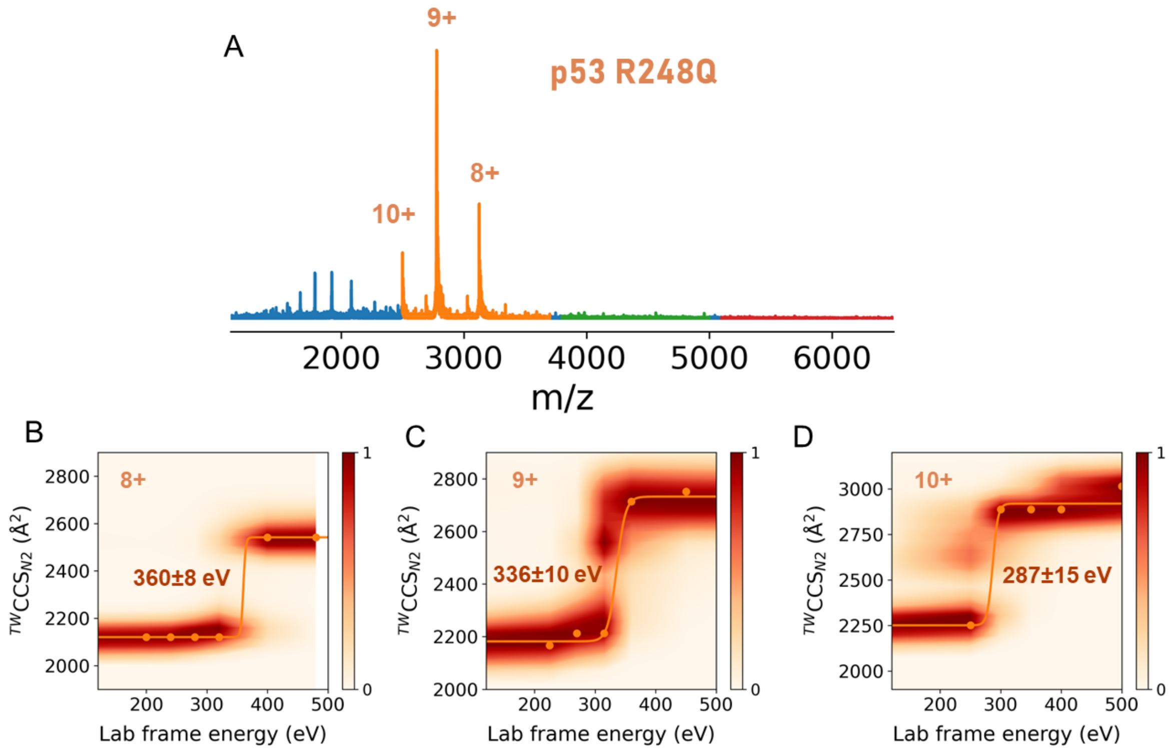

Figure S2. Native mass spectrum of R248Qp53 mutant (A) and collision-induced unfolding (CIU) heatmaps for mass-selected 8+, 9+ and 10+ ions.

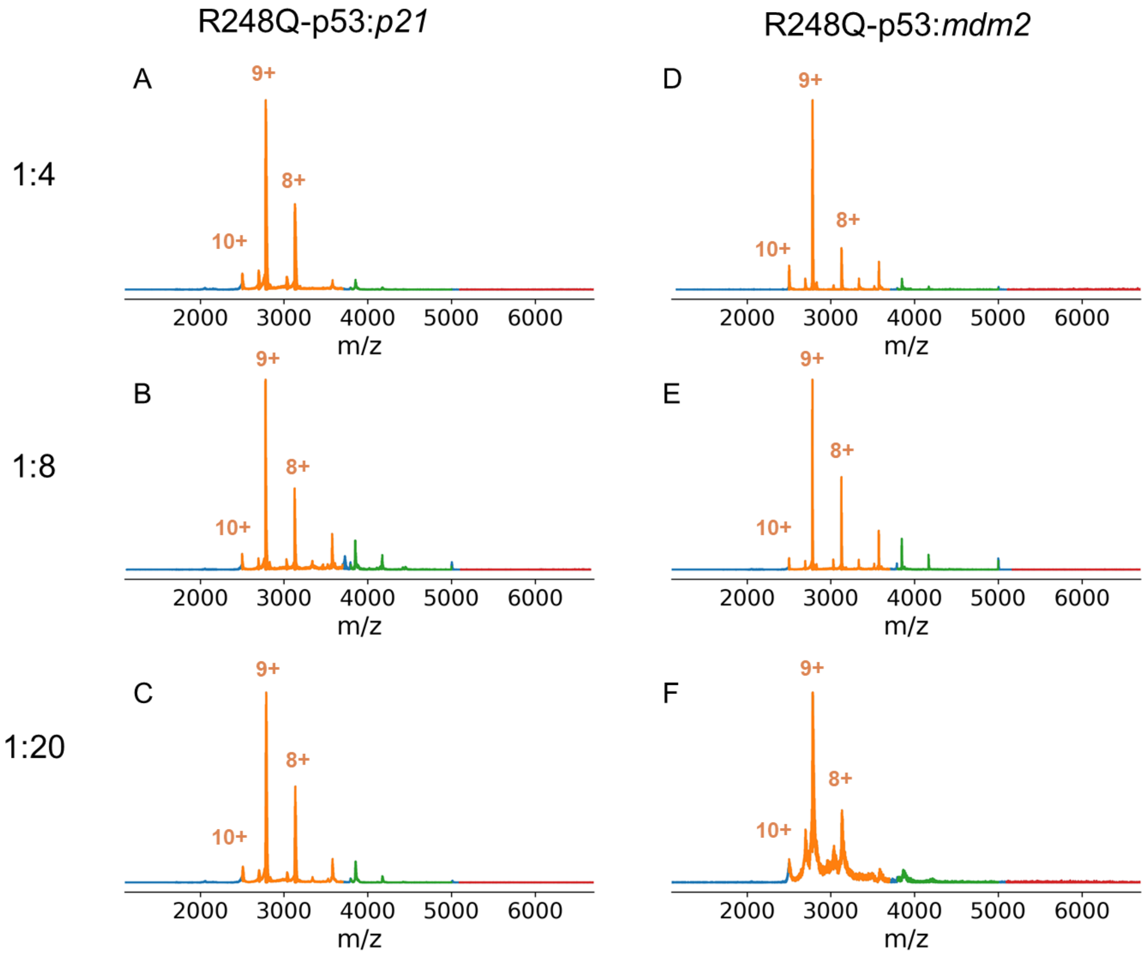

Figure S3. Native mass spectrum of R248Qp53 mutant incubated with different stoichiometries of two response elements, *p21* and *mdm2*. The 1:4, 1:4 and 1:20 refer protein to DNA ratio.

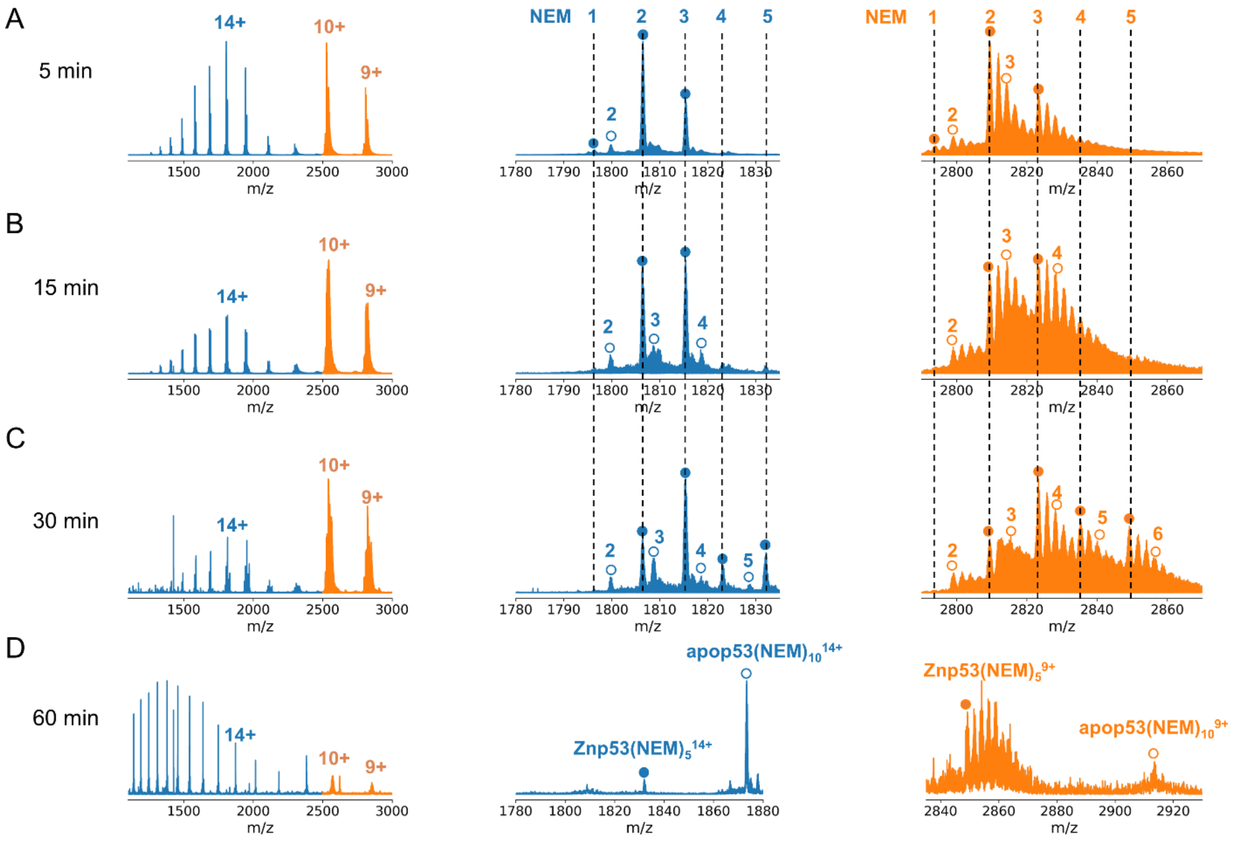

Figure S4. Native mass spectrum of WTp53 incubated for 5 (A), 15 (B), 30 (C) and 60 min (D) with 0.5 mM NEM. The middle and right colums show the species formed in the m/z regions that correspond to 14+ and 9+ ions, respectively. The full and empty circles denote holo- and apo-p53 species.

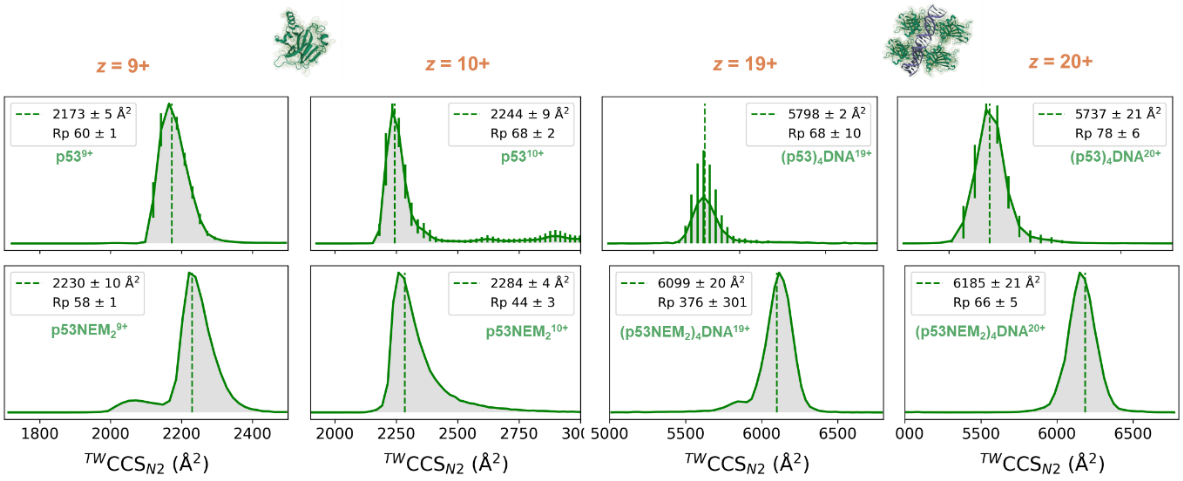

Figure S5. IM-derived CCS distributions of WTp53 incubated with 0.25 equivalents of *p21* before (A-D) and after incubation with 0.5 mM NEM (E-H) for different charge states.

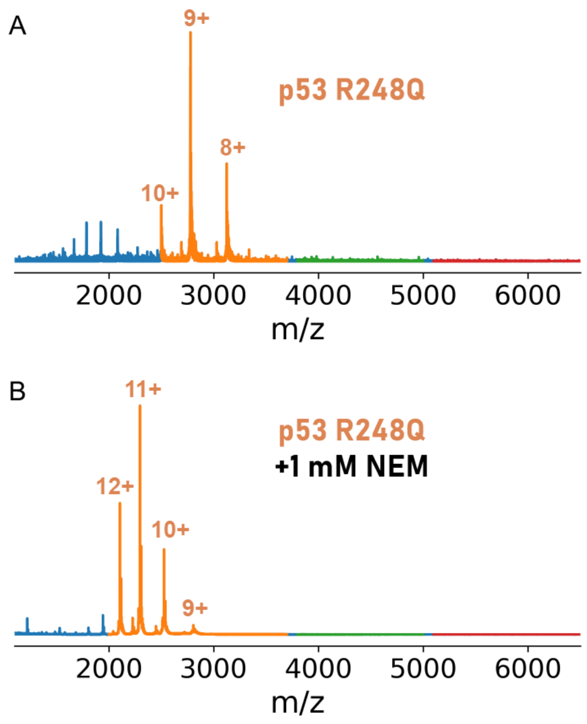

Figure S6. Native mass spectrum of R248Qp53 mutant before (A) and after incubation with 1 mM NEM (B).

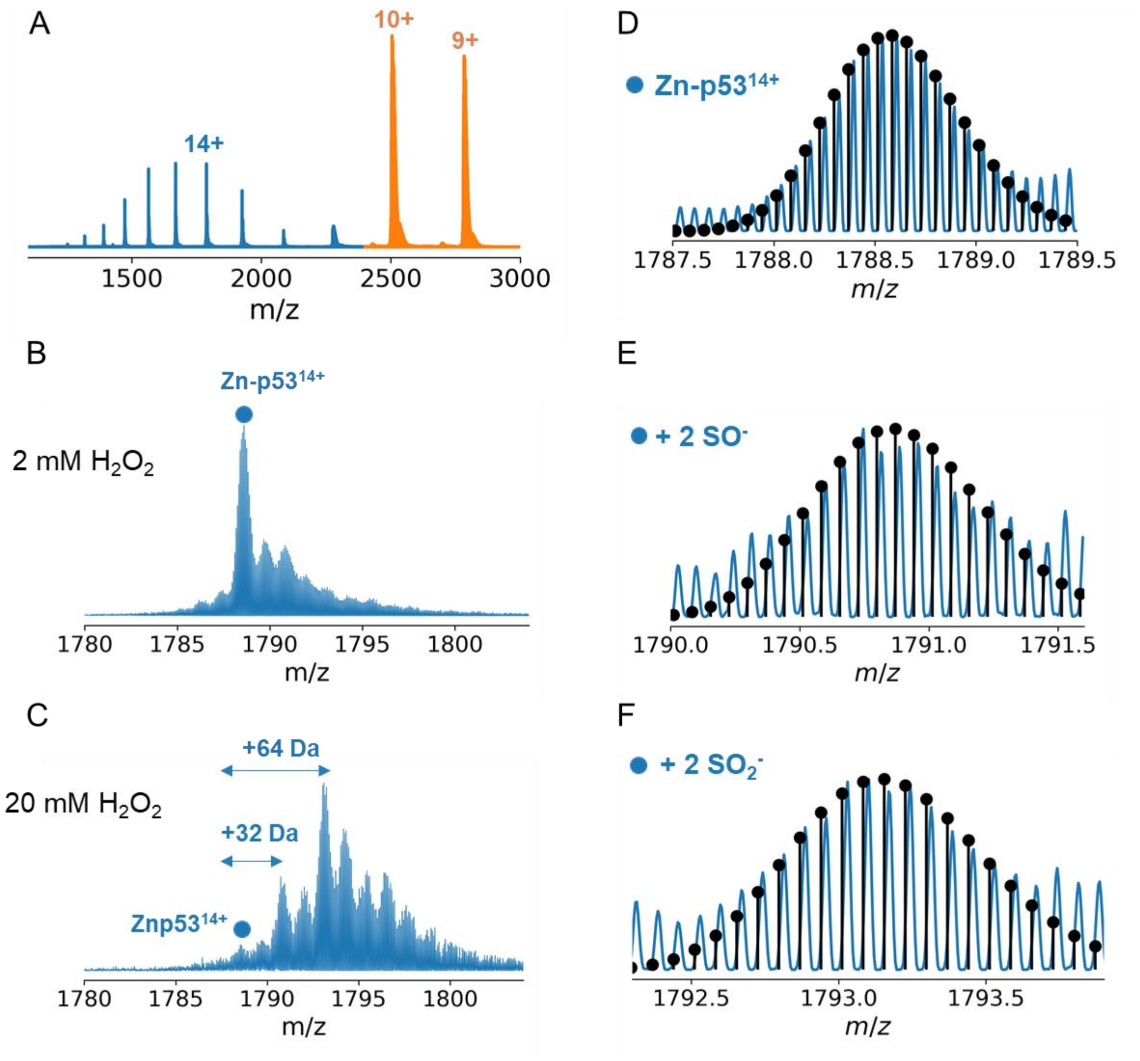

Figure S7. Native mass spectrum of WTp53 (10 µM, 200 mM ammonium acetate) incubated with 2 mm H_2O_2 (5 min, 25ºC) shows a shift in the charge state distribution toward lower charge ions (A). Species formed in the m/z region that correspond to 14+ ions when the protein was incubated with 2 mM H_2O_2 (B) and 20 mM H_2O_2 (C). Simulations of theoretical isotopic patterns for individual species were plotted as stem plots (D-F).

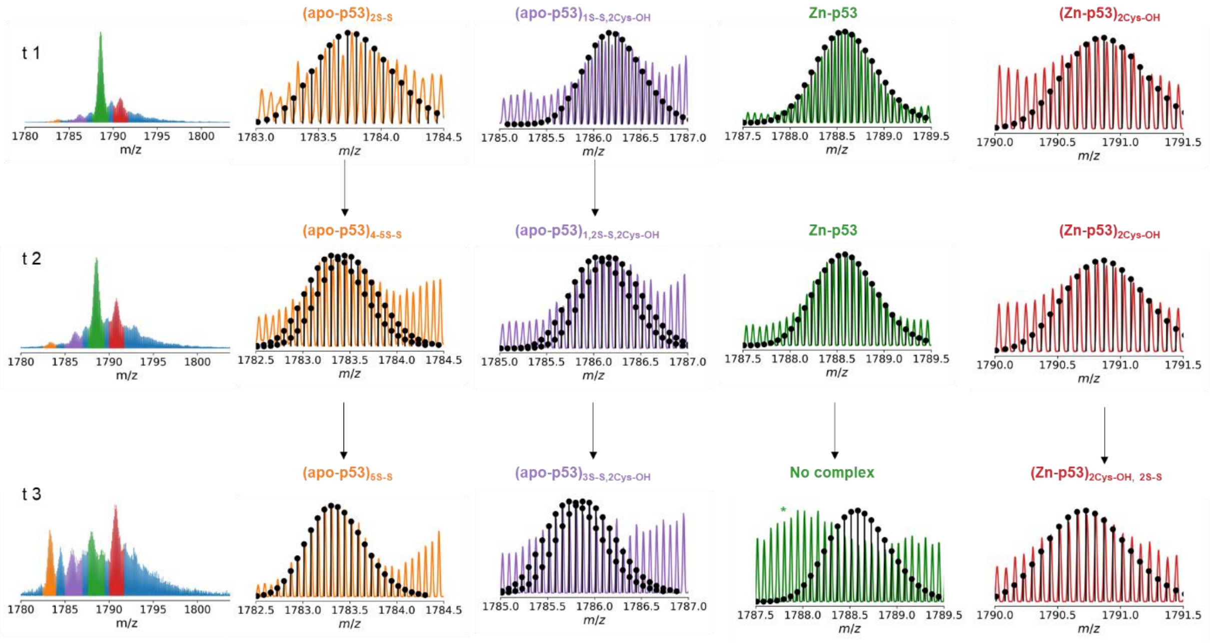

Figure S8. Extracted species formed in the m/z region that correspond to 14+ ions from the native mass spectrum of WTp53 incubated with 2 mM H_2O_2 and monitored over the time. The presence of four species were followed over the time by simulating theoretical isotopic patterns and fitting to the experimental data.

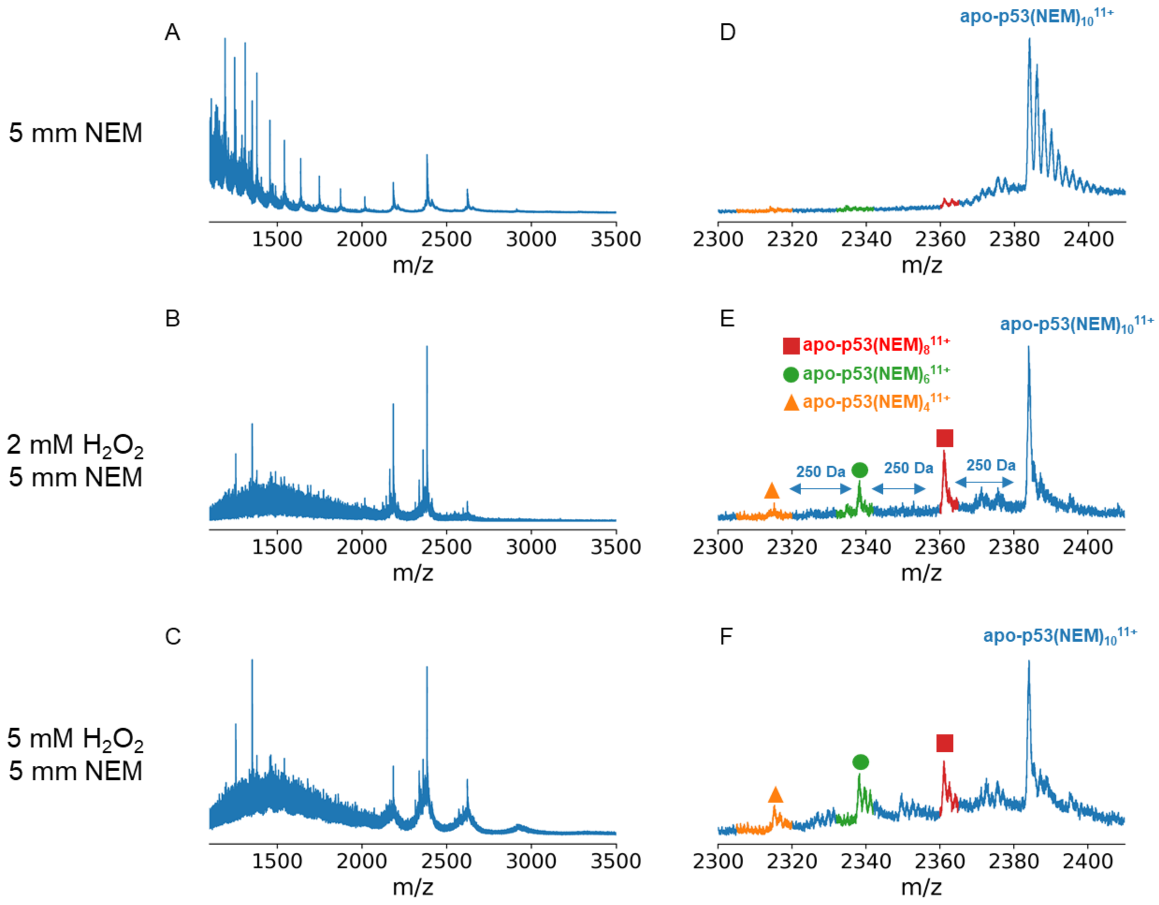

Figure S9. WTp53 was incubated with 5 mM NEM (15 min, 25ºC, dark) and analyzed under denaturing MS conditions (A). WTp53 was first incubated with 2 or 5 mM H_2O_2 (5 min, 25ºC) and then 5 mM NEM was added and incubated for 15 min in 25ºC, dark. (B and C, respectively). Extracted species formed in the 11+ m/z region that correspond to A, B and C (D, E and F, respectively).

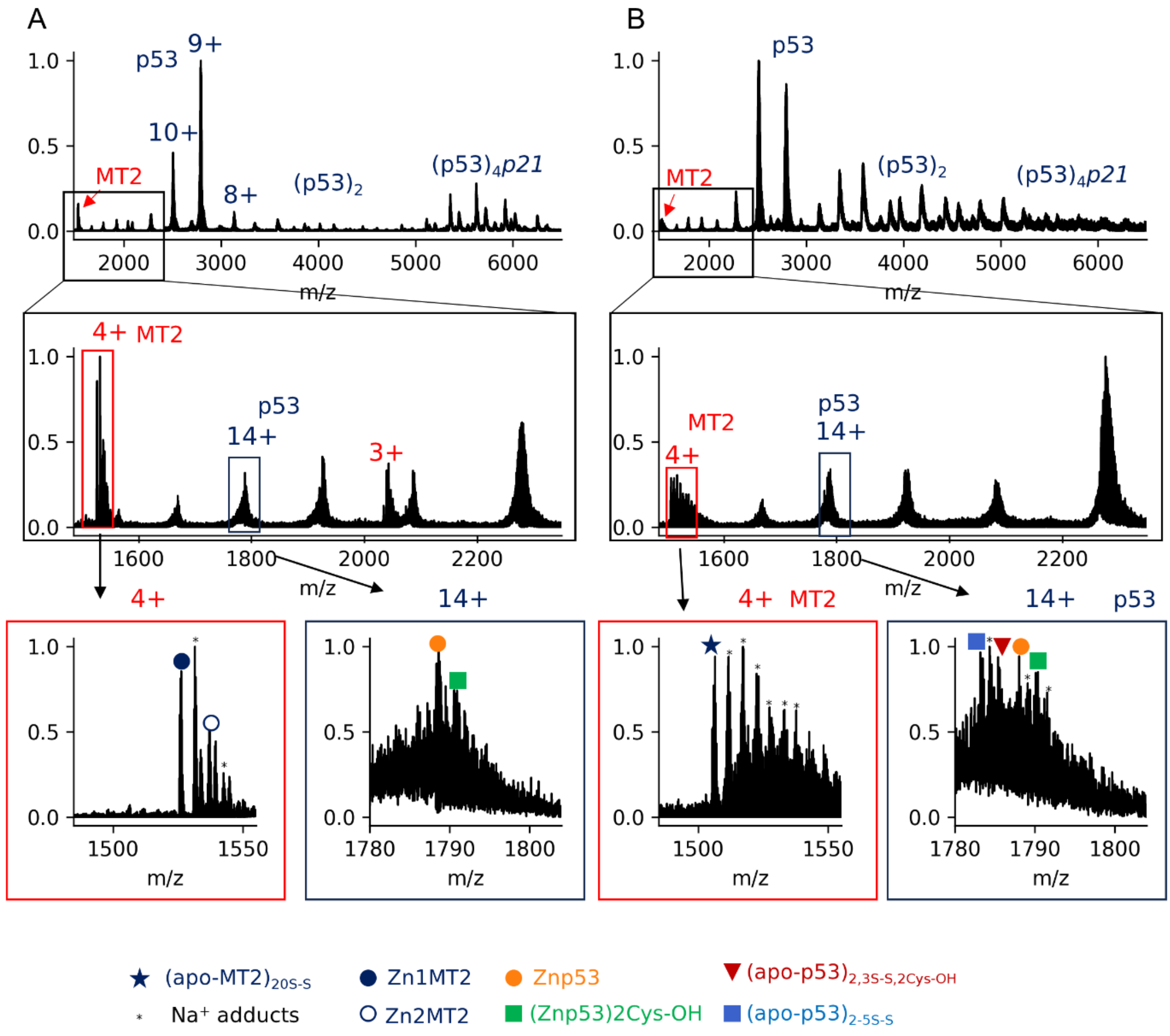

Figure S10. Native mass spectrum of WTp53 pre-incubated with 0.25 equivalents of *p21* RE, and then incubated with 20 µM of Zn_7MT2 and 2 mM H_2O_2 after 2 min (A) and 10 min (B) of reaction. The insets show the charge states corresponding to MT2 and to p53.

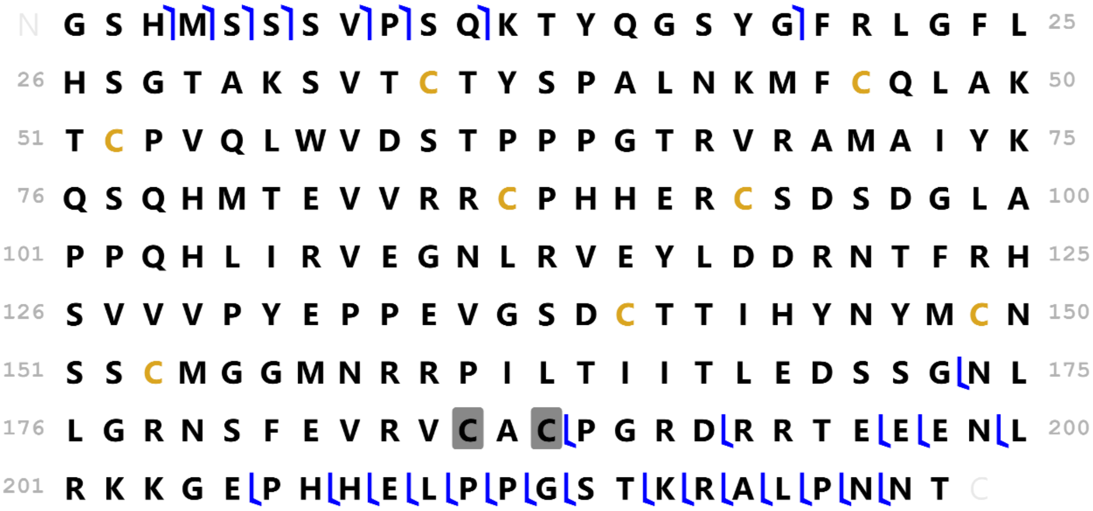

Figure S11. Fragment location map from native top-down MS analysis of the p53 reaction with H_2O_2 and incubated with dimedone. The Cys277 and Cys275 were annotated with hydrogen loss.

Table S1. Masses of species observed in the spectra shown in Figs. 1 and 2.

**Experimental mass (Da)**	**Theoretical mass (Da)**	**Assignments**
25 026.1 ± 0.3	25 026.3	p53
62 284.1 ± 0.5	50052.2(p53 dimer)<br>+12233 (double stranded p21)<br>= 62 285.2	$(p53)_2$*p21*
112 346.8 ± 0.6	112 337.4	$(p53)_4$*p21*
25 153.9 ± 0.8	25 151.1	$p53NEM_1$
25 276.8 ± 0.4	25 276.6	$p53NEM_2$
25 401.5 ± 0.2	25 401.7	$p53NEM_3$
25 526.4 ± 0.6	25 526.8	$p53NEM_4$
62 667 ± 2	62 661	$(p53NEM_2)(p53NEM_1)$*p21*
62 784 ± 1	62 786.1	$(p53NEM_2)_2$*p21*
62 911± 1	62 911.2	$(p53NEM_2)(p53NEM_3)$*p21*
113 080 ± 3	113 088.8	$(p53_4NEM_6)$*p21*
113 213 ± 3	113213.9	$(p53_4NEM_7)$*p21*
113 345 ± 4	113 339.2	$(p53NEM_2)_4$*p21*
113 483 ± 4	113 464.1	$(p53_4NEM_9)$*p21*
113 613 ± 9	113 589.2	$(p53_4NEM_{10})$*p21*
113 744 ± 5	113 714.3	$(p53_4NEM_{11})$*p21*

Table S2. Collision cross sections (CCS) of 10 µM p53 in complex with p21 in 200 mM ammonium acetate.

Species	m/z	Average $^{TW}CCS_{N2}$ (Å^2)	Standard deviation (Å^2)	$\Delta^{TW}CCS_{N2}$ (Å^2)
$[p53+8H]^{8+}$	3130.01	2119	3	26
$[p53+9H]^{9+}$	2781.25	2173	5	36
$[p53+10H]^{10+}$	2504.10	2244	9	33
$[p53+11H]^{11+}$	2276.28	2210	7	35
$[(p53)_4DNA+18H+K]^{19+}$	5913.9	5690	19	67
$[(p53)_4DNA+19H+K]^{20+}$	5618.22	5737	21	74
$[(p53)_4DNA+20H+K]^{21+}$	5350.64	5768	33	71

REFERENCES

[1] A. Ayed, F. A. Mulder, G. S. Yi, Y. Lu, L. E. Kay, C. H. Arrowsmith. Latent and active p53 are identical in conformation. *Nat. Struc. Biol*. **2001**, *8*, 756–760.

[2]A. Krężel, R. Latajka, G. D. Bujacz, W. Bal. Coordination properties of tris(2-carboxyethyl)phosphine, a newly introduced thiol reductat, and its oxide. *Inorg. Chem.* **2003**, *42*, 1994–2003.

[3] A. Kocyła, A. Pomorski, A. Krężel. Molar absorption coefficients and stability constants of metal complexes of 4-(2-pyridylazo)resorcinol (PAR): Revisiting common chelating probe for the study of metalloproteins. *J. Inorg. Biochem.* **2015**, *152*, 82–92.

[4] M. D. Peris-Diaz, R. Guran, O. Zitka, V. Adam, A. Krężel. Metal- and affinity-specific dual labeling of cysteine-rich proteins for identification of metal-binding sites. *Anal. Chem.* **2020**, *92*, 12950–12958.
