# Supplementary material for: Deciphering the safeguarding role of cysteine residues in p53 against H2O2-induced oxidation using high-resolution native mass spectrometry
Source: Commun Chem. 2025 Jan 15;8:13. doi: 10.1038/s42004-024-01395-w (PMC11736120; doi:10.1038/s42004-024-01395-w)
Supplement: Supplementary file 3 — Reporting Summary [file 42004_2024_1395_MOESM3_ESM.pdf]

nature portfolio

Corresponding author(s): Perdita Barran
Manuel Peris-Diaz

Last updated by author(s): Dec 9, 2024

Reporting Summary

Nature Portfolio wishes to improve the reproducibility of the work that we publish. This form provides structure for consistency and transparency in reporting. For further information on Nature Portfolio policies, see our [Editorial Policies](#) and the [Editorial Policy Checklist](#).

Statistics

For all statistical analyses, confirm that the following items are present in the figure legend, table legend, main text, or Methods section.

n/a	Confirmed	
☐	☒	The exact sample size (*n*) for each experimental group/condition, given as a discrete number and unit of measurement
☒	☐	A statement on whether measurements were taken from distinct samples or whether the same sample was measured repeatedly
☒	☐	The statistical test(s) used AND whether they are one- or two-sided<br>*Only common tests should be described solely by name; describe more complex techniques in the Methods section.*
☒	☐	A description of all covariates tested
☒	☐	A description of any assumptions or corrections, such as tests of normality and adjustment for multiple comparisons
☐	☒	A full description of the statistical parameters including central tendency (e.g. means) or other basic estimates (e.g. regression coefficient) AND variation (e.g. standard deviation) or associated estimates of uncertainty (e.g. confidence intervals)
☒	☐	For null hypothesis testing, the test statistic (e.g. *F*, *t*, *r*) with confidence intervals, effect sizes, degrees of freedom and *P* value noted<br>*Give P values as exact values whenever suitable.*
☒	☐	For Bayesian analysis, information on the choice of priors and Markov chain Monte Carlo settings
☒	☐	For hierarchical and complex designs, identification of the appropriate level for tests and full reporting of outcomes
☒	☐	Estimates of effect sizes (e.g. Cohen's *d*, Pearson's *r*), indicating how they were calculated

Our web collection on [statistics for biologists](#) contains articles on many of the points above.

Software and code

Policy information about [availability of computer code](#)

Data collection	MS tune QE-UHMR 2.13,MassLynx v4.2
Data analysis	MassLynx v4.2, ProSight Lite v4.1, USCF Chimera 1.13.1, Python 3.5, UniDec 5.1.1, Propka 2.0, ProteinLynx Global Server 3.0.3, imscAL19, ORIGAMI v1.2.1.6, CIUSuite 2

For manuscripts utilizing custom algorithms or software that are central to the research but not yet described in published literature, software must be made available to editors and reviewers. We strongly encourage code deposition in a community repository (e.g. GitHub). See the Nature Portfolio [guidelines for submitting code & software](#) for further information.

Data

Policy information about [availability of data](#)

All manuscripts must include a [data availability statement](#). This statement should provide the following information, where applicable:

- Accession codes, unique identifiers, or web links for publicly available datasets
- A description of any restrictions on data availability
- For clinical datasets or third party data, please ensure that the statement adheres to our [policy](#)

Processed datasets that support our results are provided in Figshare repository (10.6084/m9.figshare.25352884).

Research involving human participants, their data, or biological material

Policy information about studies with human participants or human data. See also policy information about sex, gender (identity/presentation), and sexual orientation and race, ethnicity and racism.

Reporting on sex and gender	No Human research participants were used in this work.
Reporting on race, ethnicity, or other socially relevant groupings	No Human research participants were used in this work.
Population characteristics	No Human research participants were used in this work.
Recruitment	No Human research participants were used in this work.
Ethics oversight	No Human research participants were used in this work.

Note that full information on the approval of the study protocol must also be provided in the manuscript.

Field-specific reporting

Please select the one below that is the best fit for your research. If you are not sure, read the appropriate sections before making your selection.

☒ Life sciences ☐ Behavioural & social sciences ☐ Ecological, evolutionary & environmental sciences

For a reference copy of the document with all sections, see nature.com/documents/nr-reporting-summary-flat.pdf

Life sciences study design

All studies must disclose on these points even when the disclosure is negative.

Sample size	For the direct injection MS experiments, an average spectrum was generated from a 3-minute acquisition to collect a representative spectrum.
Data exclusions	No data was excluded for the analysis.
Replication	For each experiment, three biological replicates were conducted, each with at least three technical replicates.
Randomization	No randomization was applicable since no organization in experimental groups.
Blinding	No blinding was applicable since no organization in experimental groups.

Reporting for specific materials, systems and methods

We require information from authors about some types of materials, experimental systems and methods used in many studies. Here, indicate whether each material, system or method listed is relevant to your study. If you are not sure if a list item applies to your research, read the appropriate section before selecting a response.

Materials & experimental systems

n/a	Involved in the study	
☒	☐	Antibodies
☒	☐	Eukaryotic cell lines
☒	☐	Palaeontology and archaeology
☒	☐	Animals and other organisms
☒	☐	Clinical data
☒	☐	Dual use research of concern
☒	☐	Plants

Methods

n/a	Involved in the study	
☒	☐	ChIP-seq
☒	☐	Flow cytometry
☒	☐	MRI-based neuroimaging

Plants

Seed stocks	No plants were used
Novel plant genotypes	No plants were used
Authentication	No plants were used
