# Supplementary material for: Deciphering the safeguarding role of cysteine residues in p53 against H2O2-induced oxidation using high-resolution native mass spectrometry
Source: Commun Chem. 2025 Jan 15;8:13. doi: 10.1038/s42004-024-01395-w (PMC11736120; doi:10.1038/s42004-024-01395-w)
Supplement: Supplementary file 1 — Transparent Peer Review file [file 42004_2024_1395_MOESM1_ESM.pdf]

This manuscript has been previously reviewed at another journal that is not operating a transparent peer review scheme. The manuscript was considered suitable for publication without further review at Communications Chemistry.
